# Differential Transcriptome Analysis Reveals Genes Related to Low- and High-Temperature Stress in the Fall Armyworm, *Spodoptera frugiperda*

**DOI:** 10.3389/fphys.2021.827077

**Published:** 2022-01-31

**Authors:** Mohammad Vatanparast, Youngjin Park

**Affiliations:** Plant Quarantine Technology center, Animal and Plant Quarantine Agency, Gimcheon, South Korea

**Keywords:** *Spodoptera frugiperda*, temperature, stress, RNA-seq, differentially expressed gene

## Abstract

The fall armyworm (FAW), *Spodoptera frugiperda*, is regarded as one of the world’s most harmful plant pests. This research examines the molecular response processes of FAW to low temperature (4°C) and high temperature (40°C) when gene expression is compared to controls (25°C). A total of 211,967 unigenes were collected, at least 14,338 of which were annotated with gene descriptions, gene ontology terms, and metabolic pathways. There were 50 Gene Ontology (GO) functional sub-groups and 21 EggNOG words as a result. Differentially expresses genes (DEGs) with log_2_FC ≥ 2 were identified and compared at various temperatures. In comparison to the 25°C treated group, we discovered 199 and 1,248 individual DEGs co-regulated at 4 and 40°C, respectively. Comparing transcriptome profiles for differential gene expression revealed a number of DEGs, including cytochrome P450, odorant binding proteins (OBPs), and immune system genes previously implicated in cold and high temperature stresses. The enrichment pathways were identified using Kyoto Encyclopedia of Genes and Genomics (KEGG) analysis, and heatmaps of similar unigenes from both treatment groups (T4 and T40) were plotted. We used quantitative reverse transcription PCR (RT-qPCR) to confirm the RNA-seq data on 10 up- and down-regulated DEGs. These findings provide a foundation for future understanding of FAW adaptation mechanisms and the underlying basis underlying the response to low and high temperatures.

## Introduction

The fall armyworm (FAW), *Spodoptera frugiperda* (J. E. Smith, 1797), (Lepidoptera: Noctuidae), is native to the American continent’s tropical and subtropical regions (Sparks, 1979), and is one of the world’s most destructive crop pests. This pest is polyphagous, and because of its diverse host range, *S. frugiperda* is one of the most dangerous pests threatening tropical annual crops (Andrews, 1980; Cruz et al., 1999). In 2018 and 2019, FAW was found in many Southeast Asian countries, including India, Thailand, Myanmar, China, the South Korea, Japan, the Philippines, Indonesia, and, most recently, Australia (Du Plessis et al., 2020). They are typically composed of two genetically distinct strains, including such rice (R-strain) and corn (C-strain) (Quisenberry, 1991; Nagoshi and Meagher, 2004).

The relationship between temperature and development rate has a significant influence on insect distribution and abundance (Tobin et al., 2003). Temperature is one of the most significant environmental variables that can affect an insect’s physiological condition, behavior, and evolution. Extreme temperatures, both high and low, are detrimental to insect development because they create an abiotic stress capable of causing abnormal biological responses (Kang et al., 2009). As a result, those that are subjected to stress temperatures (low and high) adopt a variety of strategies to cope with the stressful situation, including avoiding extreme temperatures, regulating the behavior of stress proteins and the oxidase mechanism, and modifying other physiological characteristics (Hoffmann et al., 2003; Sørensen et al., 2003). In overwintering insects, low-temperature stress causes a decrease in respiratory rate, reduced aerobic respiration, and damage to the cell membrane system, resulting in membrane lipid peroxidation (Sargis and Subbaiah, 2006). Energy generation, sugar, alcohol, and amino acid synthesize from fat metabolism and the tricarboxylic acid cycle (TCA cycle) will be resulted to allowing these insects to resist cold environments (Lee et al., 1991; Zhu et al., 2016, 2017). Furthermore, insects establish cold resistance using mechanisms such as freeze tolerance, freeze-avoidance and polyol accumulation (Salin et al., 2003). High-temperature stress, on the other hand, causes the presence of physiological functions in insects that change the cell microenvironment, such as cell structure and enzyme activity, as well as the spatial conformation of biological macromolecules. As a result, normal development, growth, and survival will be affected (Prange, 1996; Holmstrup et al., 2002). Many genes, including those encoding heat shock proteins (HSPs), heat shock transcription factors, the hsr-omega protein, and phosphoglucose isomerase, are upregulated in response to high temperature stress in insects (Solomon et al., 1991; Morgan and Mackay, 2006; Rank et al., 2007; Chen et al., 2016).

Fall armyworm can withstand high temperatures because it is native to the tropics and subtropics (Sparks, 1979). Since FAW lacks a diapause function, it can only live in tropical and subtropical areas during the winter in America and elsewhere, limiting its geographic range and ability to adapt to cold temperatures (Dan-dan et al., 2020). After exposing all life stages of FAW to low temperatures for 3 h, it was revealed that the egg was the most resistant stage, with 30% survival at −10°C (Foster and Cherry, 1987).

Recently, omic technologies, such as *de novo* transcriptome assembly, have been commonly used to detect and classify differential genes under various experimental conditions (He et al., 2017; Liu et al., 2017; Chen et al., 2018; Li et al., 2020) and revealed how environmental physiologists explore stress response pathways in insects (Zhou et al., 2019). Transcriptome analysis using RNA-seq has been used to investigate gene expression changes in response to thermal stress in several insect species since 2014; *Drosophila virilis* (Parker et al., 2015), *Cryptolaemus montrouzieri* (Zhang et al., 2015b), *Microdera punctipennis* (Tusong et al., 2016), *Nilaparvata lugens*, *Sogatella furcifera*, *Laodelphax striatellus* (Huang et al., 2017), *Galeruca daurica* (Zhou et al., 2019), and *Monochamus alternatus* (Li et al., 2020). These studies concluded that cold stress can alter the expression levels of hundreds of genes involved in transcription, metabolism, and cuticular organization, especially enzyme-related genes, as well as high-temperature stress, which upregulates encoding cytochrome P450s (P450), antioxidative enzymes, and aldehyde dehydrogenase (Wei et al., 2015; Zhang et al., 2015b; Liu et al., 2017). HSPs have recently been confirmed to be responsible for heat and cold tolerance as well as other stressors (e.g., heat, low oxygen levels, UV radiation, bacterial and viral infection, and heavy metals) that can affect the folding and functional conformation of proteins (Clark and Worland, 2008; Storey and Storey, 2012).

In the present work, we used RNA-seq and *de novo* transcriptome assembly to produce transcriptomes and analyze the changes in transcription regulation associated with cold and heat treatment in *S. frugiperda*. A detailed differential expression analysis revealed a number of candidate genes that may be associated with FAW cold and heat tolerance. To validate the RNA-seq results, we used quantitative reverse transcription PCR (RT-qPCR). We hoped to provide a foundation for the adaptive mechanism as well as a rich resource for the discovery and detection of novel genes involved in cold and heat stress responses of *S. frugiperda*.

## Materials and Methods

### Insect Rearing, Exposure Temperatures, and Sample Preparation

The larvae of *S. frugiperda* (F0 generation) were obtained from Frontier Agriculture Sciences (Newark, DE, United States). They were raised until pupation under laboratory-controlled conditions [26°C ± 1, 70% ± 5 RH, and a photoperiod of 14 h:10 h (L:D)]. The larvae were fed an artificial diet (Newark, DE, United States) during their development (Vatanparast et al., 2020). The diet was changed every day for larvae. These larvae were grown in plastic containers with aerated lids measuring 40 × 20 × 15 cm. From the third instar onward, larvae were reared separately to prevent cannibalism. This was carried out in Petri dishes (8.5 cm diameter). Pupae were sexed and kept with the larvae in the same place. Pupae were monitored on a regular basis before moths appeared. Fourth instar larvae were incubated at 4 and 40°C for 16 h as temperature treatment groups to conduct transcriptomic analysis. The control group consisted of larvae incubated at 25°C. Based on a prior work on *Spodoptera exigua*, the temperature and incubation time were chosen (Park and Kim, 2013). *S. frugiperda* also exhibits rapid cold hardening at 4°C, according to our findings (data was not published). A total of 30 larvae were considered for each temperature. The larvae from each group were immediately frozen in liquid nitrogen and preserved at −80°C for subsequent experiments after the temperature procedure.

### RNA Extraction and Quantitative Reverse Transcription PCR

For this study, total RNA was isolated from five *S. frugiperda* larvae bodies (fifth instar) with the help of TRIzol reagent (Invitrogen; Carlsbad, CA, United States) (Vatanparast et al., 2020). Nuclease-free water was used for RNA extraction, and spectrophotometers were used to measure its concentration (NanoDrop, Thermo Scientific, Wilmington, DE, United States). As instructed by the manufacturer, we used Intron Biotechnology’s Intron PreMix (Seoul, South Korea) to synthesis cDNA from one microgram of RNA. The CFX Connect Real-Time PCR Detection System (Bio-Rad, Hercules, CA, United States) and iQ SYBR Green Supermix (Bio-Rad, Hercules, CA, United States) were used to perform all RT-qPCR in this investigation. Each forward and reverse primer (Supplementary Table 1) was added to the reaction mixture, as well as the nuclease-free water, to make 20 μL of total reaction volume. Temperature cycling for RT-qPCR began with a 10-min heat treatment at 95°C followed by 40 cycles of denaturation for 30 s, annealing for 30 s, and extension for 20 s each at 72°C. Use of the EF1α gene expression level to normalize the expression levels of the target genes was done (Shu et al., 2021) under different treatments. Melting curve analysis was used to evaluate the PCR products. Each treatment was replicated with three independent biological sample preparations. The comparative CT approach (2^–ΔΔCT^) was used for quantitative analysis (Livak and Schmittgen, 2001).

### Illumina Sequencing

Illumina sequencing was performed on Macrogen to achieve short-read RNA sequences (Seoul, South Korea). The TruSeQ Stranded MRNA LT Sample Prep Kit (Illumina, San Diego, CA, United States) has been used to build each library with a 1 μg total RNA of the whole group of 5 individuals *S. frugiperda* larvae per treatment and with the 101 bp pair end reading HiSeq 4000 System (Illumina, San Diego, CA, United States), it was sequenced (Supplementary Table 2).

### *De novo* Assembly

Illumina short reads were quality-filtered and adapter-trimmed using Trimmomatic v0.38.^1^ FastQC v0.11.7^2^ was used to check data quality before and after trimming. After the removal of low-quality reads, an Illumina-based *de novo* transcriptome assembly was performed using Trinity version trinity rnaseq r20140717, bowtie 1.1.2 (Langmead et al., 2009). Trimmed reads for every sample were merged into one file to construct combined reference. The *de novo* assembly of merged data was carried out using Trinity with default parameters that it was assembled into transcript contigs (Grabherr et al., 2011). Total number of genes, transcripts, GC content max/min/median/average contig length and total assembled bases were summarized. Trinity groups transcripts into clusters based on shared sequence content. For assembled genes, longest contigs of the assembled contigs are filtered and clustered into the non-redundant transcripts using CD-HIT version 4.6^3^ (Pertea et al., 2003). These transcripts were defined as “unigenes” which are used for predicting the ORFs (open reading frames), annotating against several known sequence databases, and analyzing differentially expressed genes (DEGs). ORF prediction for unigenes was performed using TransDecoder version 3.0.1^4^ (Haas et al., 2013) to identify candidate coding regions within transcript sequence. After extracting ORFs that were at least 100 amino acids long, TransDecoder predicted the likely coding regions. Trimmed reads for each sample were aligned to the assembled reference using Bowtie program. For the DEG analysis, the abundances of unigenes across samples were estimated into read count as an expression measure by RSEM algorithm (RSEM version v1.2.29, bowtie 1.1.2^5^; Li and Dewey, 2011).

### Gene Functional Annotation

For functional annotation, unigenes were searched against Kyoto Encyclopedia of Genes and Genomics (KEGG) v20190104^6^ (Kanehisa et al., 2004), NCBI Nucleotide (NT) v20180116^7^ (Zhou et al., 2019), Pfam v20160316^8^ (Finn et al., 2011), Gene Ontology (GO) v20180319^9^ (Ashburner et al., 2000), NCBI non-redundant Protein (NR) v20180503^10^ (Deng et al., 2006), UniProt v20180116^11^ (Apweiler et al., 2004), and EggNOG^12^ (Powell et al., 2013) using BLASTN of NCBI BLAST and BLASTX of DIAMOND version 0.9.21^13^ with an *E*-value default cutoff of *E*-value 10^–5^.

### Differential Gene Expression Analysis

Quality check was done for all samples, so that if more than one read count value was 0, it was not included in the analysis. In order to reduce systematic bias, estimates the size factors from the count data and applies Relative Log Expression (RLE) normalization with DESeq_2_ R library. Using each sample has normalized value, the high expression similarities were grouped together by Hierarchical Clustering Analysis and graphically shown in a 2D plot to show the variability of the total data using Multidimensional Scaling Analysis. Significant unigene results were analyzed as up and down-regulated count by log_2_FC ≥ 2 and ≤−2, distribution of expression level between two groups was plotted as volcano plot^14^ (Goedhart and Luijsterburg, 2020) and simple bar plots. Heat maps were generated using the online tool Heatmapper^15^ (Babicki et al., 2016). The KEGG analysis were done to identify the enrichment pathways and heat maps of shared unigenes at both treatment groups (T4 and T40) in comparison with T25 were plotted. In these cases, to get more effective DEGs, the DEGs were limited to log_2_FC ≥ 5 and ≤ −5, with a *P*-value < 0.01.

### Quantitative Reverse Transcription-PCR Validation

Quantitative reverse transcription PCR was used to verify the 10 genes that respond to low (T4) and high (T40) temperatures. Separate sets of 10 FAW larvae each were incubated at 4 and 40°C for 16 h in order to perform the experiment successfully. In accordance with section “RNA Extraction and Quantitative Reverse Transcription PCR,” the cDNA was synthesized. Primer Quest^16^ was used to create custom primers (Supplementary Table 8). A gene called EF1α was employed as a reference gene to standardize the expression levels of target genes under various treatments (Shu et al., 2021). Finally, the data was compared based on the fold change (FC) ratio and the mRNA expression level ratio for all identified genes (Vatanparast et al., 2021).

### Statistical Analysis

All of the studies were carried out in three biological replicates. GraphPad Prism 8.0 was used to plot the results. Means were compared using the least squared difference (LSD) test of one-way analysis of variance (ANOVA) in the SAS program (Sas Institute Inc, 1989; Vatanparast and Park, 2021), with Type I error = 0.05 as the threshold for discrimination.

## Results

### Sequencing, RNA-Seq Assembly, and Functional Annotation

Filtering of Illumina raw data revealed transcriptome responses to low and high stress temperature in *S. frugiperda* (Supplementary Tables 3, 4). After transcriptome sequencing of cDNA samples, 19.2 Gb of clean data passed the Illumina quality filter with Q30 > 95% (Supplementary Table 4). All high-quality reads (Supplementary Table 4) were pooled to perform the *de novo* transcriptome assembly. NGS data of *S. frugiperda* used in this study has been deposited in the NCBI database (accession No. GSE175545). These contigs were further assembled into 227,950 transcripts with a mean length of 391.0 bp and a N50 of 1,021 bp, and 211,967 unigenes with a mean length of 356.0 bp and a N50 of 853 bp using paired-end joining and clustering based on contig similarity (Supplementary Tables 5, 6). The length distribution of unigenes closely followed the length distribution of transcripts. This indicates a high-quality assembly, providing a sequence basis for future studies.

### Annotation of Predicted Proteins

BLASTX was used to validate and annotate the assembled unigenes against ten public databases (NR, NT, UniProt, Pfam, GO, EggNOG, KEGG, KO EUK Annotation, KO PRO Annotation, KO BAC Annotation, and KO BAC.NUC Annotation) with a cut off *E*-value of 10^–5^. Genes with a large blast hit to arthropods were detected after annotation. Total of 33,669 (15.9%) unigenes were found in the NT public database, followed by the NR database (27,227 annotated unigenes, 12.87%) and the KO EUK Annotation database (25,617, 12.09%) (Table 1). Overall, the majority of the unigenes were either unable to be annotated or had inadequate definitions (e.g., putative, unknown, hypothetical, or unnamed protein). However, BLASTX matches in the NT database revealed that the unigene sequences were most similar to gene sequences from *Spodoptera litura* (74.25%) and had a total of more than 96.2% similarity with lepidopteran genus (*Spodoptera* sp., *Helicoverpa* sp., *Bombyx* sp., *Amyelois* sp., *Papilio* sp., *Plutella* sp., and *Pieris* sp.).

**TABLE 1:** Statistics of annotation analysis of unigenes.

Database	Unigene (%)	300 ≤ length < 1,000	Length ≥ 1,000
NT_Annotation	33,699 (15.9)	13,665	13,027
NR_Annotation	27,277 (12.87)	10,080	11,635
Pfam_Annotation	16,504 (7.79)	5,128	8,986
EggNOG_Annotation	24,077 (11.36)	8,560	11,045
KO_EUK_Annotation	25,617 (12.09)	9,336	11,357
GO_Annotation	16,279 (7.68)	4,884	9,243
UniProt_Annotation	14,338 (6.76)	4,127	8,428

The TransDecoder software was used to predict ORFs for unigenes. ORFs of at least 100 amino acids in length were isolated. At least one ORF was found in 94.73% (12,567) of the total expected unigenes (211,967) (Table 2). Of the 14,052 expected ORFs, 7,918 (56.35%) were found to be completely ORFs (Table 2).

**TABLE 2:** Statistics of open reading frame (ORF) prediction.

Assembly	Total unigene	ORF predicted unigene	Single ORF predicted unigene	Multiple ORF predicted unigene	
Merge	211,967	13,276 (6.26%)	12,576 (94.73%)	700 (5.27%)	

**Assembly**	**Number of ORF**	**Complete**	**Internal**	**5′ partial**	**3′ partial**

Merge	14,052	7,918 (56.35%)	2,208 (15.71%)	2,174 (15.47%)	1,752 (12.47%)

### EggNOG Analysis for Global Functional Classification

**FIGURE 1:**
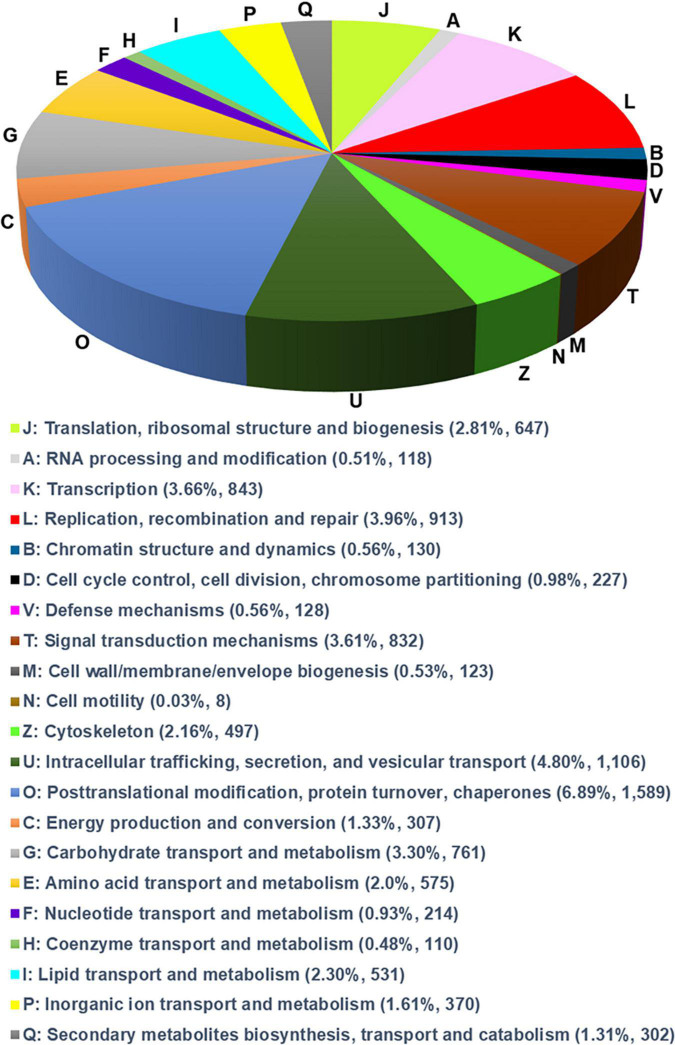
Annotations of the annotated unigenes. The annotated unigenes are mapped to the annotation of the corresponding orthologous groups in EggNOG (evolutionary genealogy of genes) database.

The EggNOG database was used to classify the annotated unigenes using BLASTX for functional annotation and to reveal functional and biological classification of the unigenes. In total, 24,077 unigenes were allocated to 21 EggNOG terms (Figure 1) from three functional classes: “information storage and processing,” “cellular processes and signaling,” and “metabolism.” The most unigenes were identified as “post-translational modification, protein turnover, chaperones (1,589 unigenes),” “intracellular trafficking, secretion, and vesicular transport (1,106),” “replication, recombination, and repair (913),” “transcription (843),” and “signal transduction mechanism (832)” (Figure 1).

### Differentially Gene Expression Under Different Temperature

**FIGURE 2:**
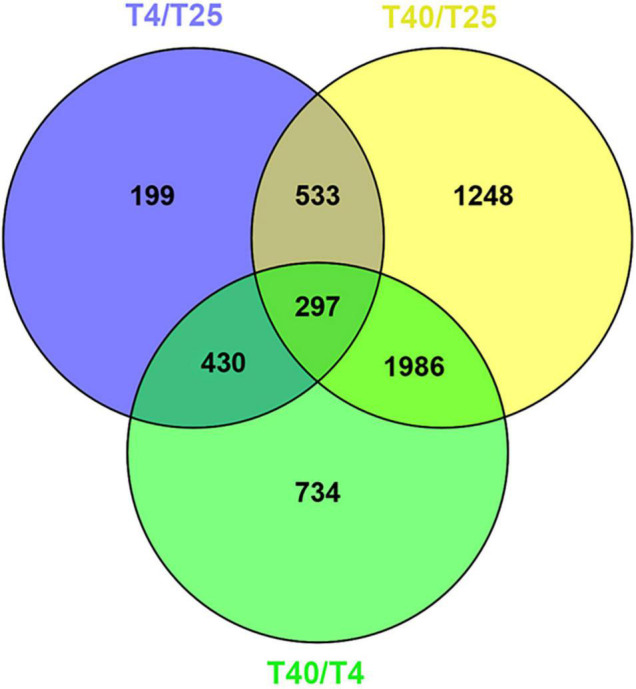
Venn diagram analysis. The number of specific and shared DEGs were showed between three different treatment groups with *P*-value < 0.05 and log_2_FC ≥ 2 or log_2_FC ≤ –2.

**FIGURE 3:**
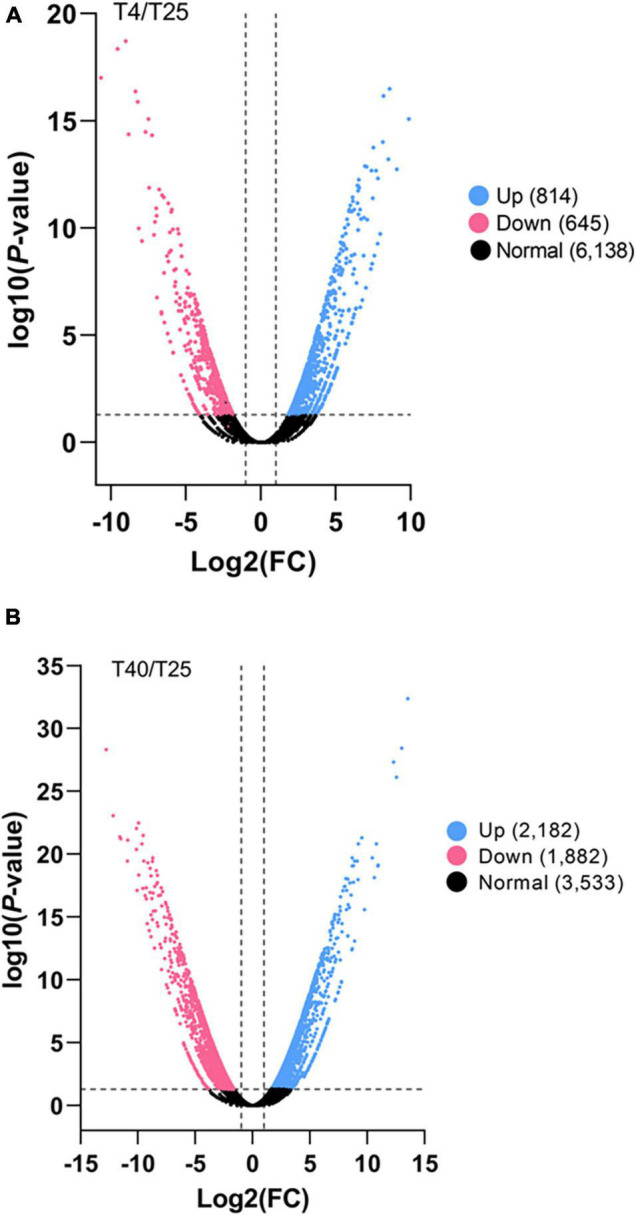
Significant differentially expressed gene (DEG) analysis. The number of all up and down regulated contigs based on *P*-value < 0.05 and log_2_FC ≥ 2 or log_2_FC ≤ –2 of comparison pairs was plotted. Volcano diagram for distribution of the identified DEGs in treatment groups **(A)** T4 and **(B)** T40, in comparison with T25 as control was plotted. Red and blue points represent the significant DEGs that down-regulated or up-regulated, respectively and black ones show those without significant difference.

Differentially expresses genes were identified in comparison to the control (T25) under low and high stress temperature treatments (T4 and T40, respectively). With a criterion of modified *P*-value < 0.05 and | log_2_FC| ≥ 2, 199 unigenes were DEGs for T4 and 1248 unigenes were DEGs for T40. In addition, 734 DEGs were found explicitly for T40 as compared to T4 (Figure 2). Volcano plots with a criterion of *P*-value < 0.05 and | log_2_FC| ≥ 2 were plotted for each treatment in comparison to T25 as the control to classify more likely particular genes linked to temperatures (Figure 3).

**FIGURE 4:**
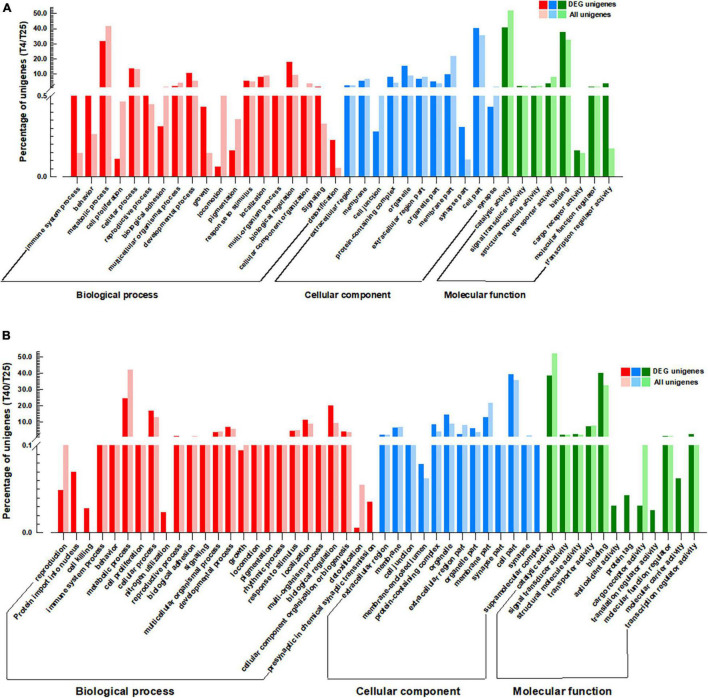
Gene Ontology (GO) analysis. GO annotations of the specific differentially expressed genes (DEGs) (*P*-value < 0.05, log_2_FC ≥ 2, ≤–2) coregulated by cold **(A)** and high **(B)** temperature stresses in comparison with T25 as control group. GO terms are summarized in three main categories of “Biological process,” “Cellular component,” and “Molecular function.” The *Y*-axis shows the percentage of unigenes in each category.

**FIGURE 5:**
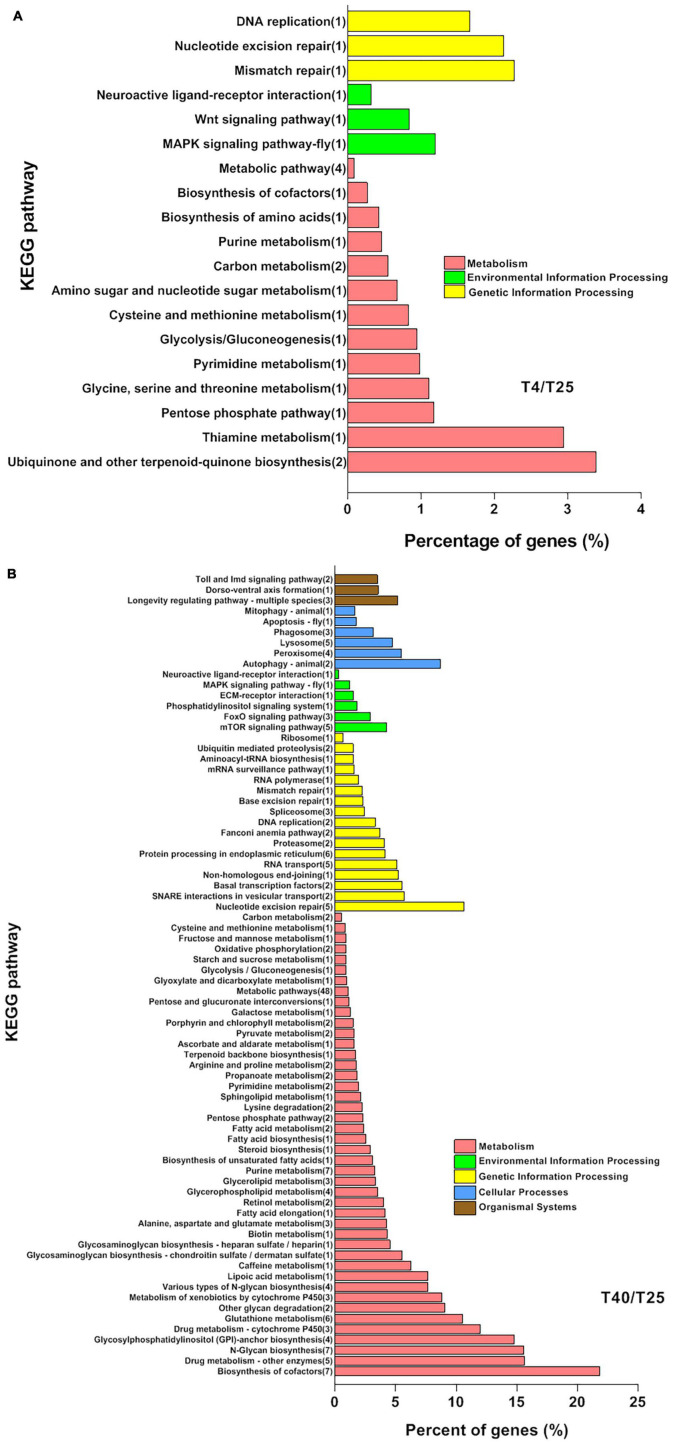
Kyoto Encyclopedia of Genes and Genomes pathways enrichment analysis. Analysis of significant KEGG pathways enrichment in the specific differentially expressed genes (DEGs) (*P*-value < 0.01, log_2_FC ≥ 5, ≤–5) coregulated by cold **(A)** and high **(B)** temperature stresses in comparison with T25 as control group. The *Y*-axis indicates the KEGG pathways, and the *X*-axis indicates the percentage of unigenes in each pathway, indicated on the left side of bars. The numbers in the brackets are DEGs significantly enriched in the relevant pathways.

For screened unigenes, GO and KEGG analyses were performed to clarify functional classification (Figures 4, 5 and Supplementary Table 7, respectively). The functions of DEGs that were substantially different (FC ≥ 2 or ≤ −2) with incubation of *S. frugiperda* larvae at 4 and 40°C in comparison to 25°C were clarified using GO annotation (Figure 4). At low temperatures, the larvae’s the most important GO terms were “Biological process: cellular process, developmental process, response to stimulus, and biological regulation,” “Cellular component: protein-containing complex, organelle, organelle part, and cell part,” and “Molecular function: binding, molecular function regulator, and transcription regulator activity” (Figure 4A). The comparison of GO data between the T40 and T25 groups revealed a significant increase in the percentage of unigenes as follows: “Biological Process: behavior, cellular process, reproductive process, developmental process, localization, biological regulation, and cellular component organization or biogenesis,” “Cellular Component: cell junction, protein-containing complex, organelle, organelle part, and cell part” (Figure 4B). The key DEG pathways were discovered using KEGG pathway enrichment research (Figure 4 and Supplementary Table 7). When compared to the T25 treatment group, both treatment groups showed pathway enrichment. As predicted, the T4 group had a lower impact on pathway enrichment than the T40 group (Figure 5A). Under cold temperature stress, four coregulated DEGs were significantly enriched in the “Metabolic pathways,” which are dominated by unigenes such as “glucose-6-phosphate isomerase,” “adenylate kinase isoenzyme 1-like isoform X1,” “D-3-phosphoglycerate dehydrogenase,” and “4-coumarate-coa ligase 1-like.” Another pathways enriched under the influence of low temperature were “Carbon metabolism,” “Ubiquinone and other terpenoid-quinone biosynthesis,” “Starch and sucrose metabolism,” “Glycolysis/Gluconeogenesis,” “DNA replication,” and “MAPK signaling pathway” (Figure 5A and Supplementary Table 7).

Based on KEGG analysis data related to larvae incubated at higher temperatures, a greater number of pathways were enriched, as expected (T40). In high temperature conditions, 219 unigenes were found to enrich different pathways. Metabolic pathways were significantly enriched by 48 unique DEGs. Along with “Fatty acid elongation,” “Fatty acid biosynthesis,” “Fatty acid metabolism,” “Starch and sucrose metabolism,” and “Fructose and mannose metabolism,” other enriched pathways connected to the “Metabolism” branch of KEGG research included “Cofactor biosynthesis,” “Purine metabolism,” and “Glutathione metabolism.” In high temperature treatments, pathways linked to “Cellular Processes” and “Organismal Systems” were found that were not present in low temperature treatments. Furthermore, as compared to the T4 treatment community, the number of pathways linked to Environmental Information Processing and Genetic Information Processing increased (Figure 5B and Supplementary Table 7). To explore the shared DEGs involved in cold and high temperatures stresses condition, we screened the same genes in with new criteria (*P*-value < 0.01 and |FC| ≥ 5). We reasoned that by introducing new criteria, we will be able to identify DEGs that are more successful.

**FIGURE 6:**
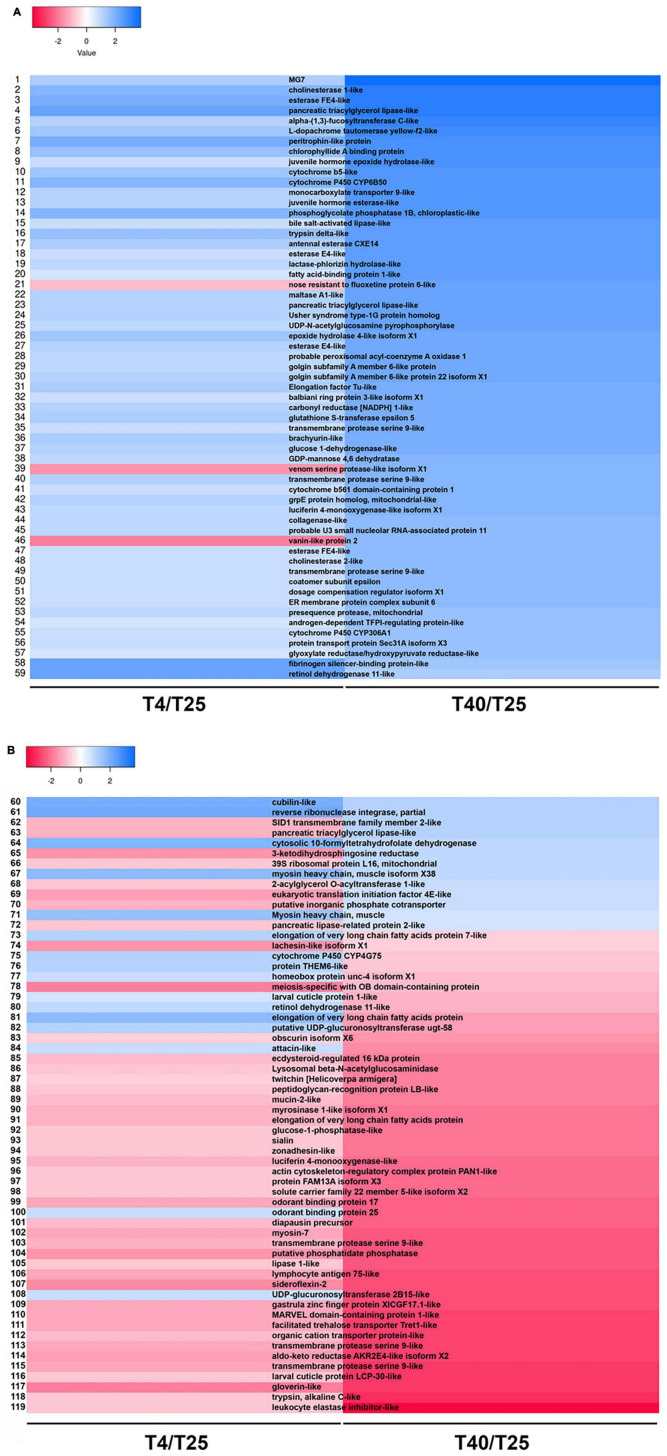
Distribution of same differentially expressed genes (DEGs) between treatment groups. **(A,B)** Heatmap shows expression patterns for same DEGs (*P*-value < 0.01, log_2_FC ≥ 5, ≤–5) involved in low (4°C) and high (40°C) temperature treatment groups in comparison with T25 as group of control.

To make the data more understandable, heat map plotting was used (Figure 6). For these plots, the log10 of FC was used. A total of 119 unigenes were discovered and plotted in two heat maps (Figures 6A,B and Supplementary Table 8). “juvenile hormone epoxide hydrolase-like,” “cytochrome b5-like,” “cytochrome P450 CYP6B50,” “juvenile hormone esterase-like,” “alpha-(1,3)-fucosyltransferase C-like,” “peritrophin-like protein,” “esterase E4-like,” “fatty acid-binding protein 1-like,” and “epoxide hydrolase 4-like isoform X1” are some DEGs that up-regulated in both group (T4 and T40) but they show significantly high level mRNA expression level in high temperature (Figure 6A and Supplementary Table 8). We found some DEGs showed upregulation in high temperatures condition but downregulated in low temperature. Some of them are including: “nose resistant to fluoxetine protein 6-like,” “venom serine protease-like isoform X1,” “vanin-like protein 2,” “SID1 transmembrane family member 2-like,” “pancreatic triacylglycerol lipase-like,” “3-ketodihydrosphingosine reductase,” “39S ribosomal protein L16, mitochondrial,” “2-acylglycerol O-acyltransferase 1-like,” “eukaryotic translation initiation factor 4E-like,” “putative inorganic phosphate cotransporter,” and “pancreatic lipase-related protein 2-like.” In contrast with previous group some DEGs upregulated in low temperature and downregulated in T40 treatment group including: “elongation of very long chain fatty acids protein 7-like,” “cytochrome P450 CYP4G75,” “glycerol-3-phosphate dehydrogenase,” “homeobox protein unc-4 isoform X1,” “larval cuticle protein 1-like,” “glycerol kinase,” “elongation of very long chain fatty acids protein,” “putative UDP-glucuronosyltransferase ugt-58,” “attacin-like,” “odorant binding protein 25,” and “UDP-glucuronosyltransferase 2B15-like” (Figure 6B and Supplementary Table 8).

### Validation of Gene Expression Profiles by Quantitative Reverse Transcription PCR

**FIGURE 7:**
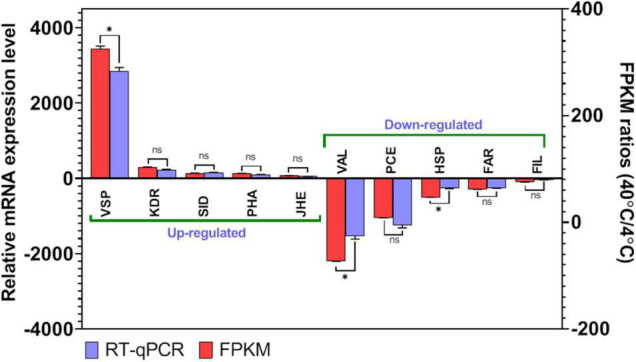
Differentially expressed genes (DEGs) validation by RT-qPCR in comparison to corresponding folding change (FC) data detected in RNA-Seq. Relative genes expression (T40/T4) is shown as the ratio of them per *EF1*α. Each treatment includes three technical replicates. Asterisks above standard deviation bars indicate significant difference between groups at Type I error = 0.05 (LSD test). The letter “ns” stands for a non-significant difference between groups.

To confirm the accuracy and reproducibility of the Illumina RNA-Seq, RT-qPCR and gel electrophoresis of 10 common DEGs from T40 in comparison to T4 group described in the RNA sequence data were performed (Figure 7 and Supplementary Table 1). Five upregulated DEGs were included “venom serine protease 34-like isoform X1” (VSP; c214749_g1_i1), “3-ketodihydrosphingosine reductase” (KDR; c170666_g1_i1), “SID1 transmembrane family member 2-like” (SID; c180292_g9_i4), “phospholipase A1-like” (PHA; c170786_g1_i1), and “juvenile hormone epoxide hydrolase-like” (JHE; c179215_g2_i2). Five other DEGs showing down-regulation in T40 in relation to T4 have been validated by RT-qPCR including; “venom allergen 5.01-like” (VAL; c166591_g1_i1), “proclotting enzyme-like” (PCE; c182964_g5_i1), “heat shock protein 68-like” (HSP; c180345_g2_i1), “fatty acyl-CoA reductase wat-like” (FAR; c179097_g1_i1), and “filamin-A” (FIL; c185519_g3_i1). It can be seen that changes in expression for the RT-qPCR are in the same direction. The Illumina sequencing results were compatible with RT-qPCR data, which check that the transcriptome analysis was reliable and accurate. Through this analysis the findings from RNA-Seq will ensure that DEGs will be identified under stressful conditions and that further investigations of these or other DEGs from the transcriptome data are feasible and sustainable.

## Discussion

A comprehensive analysis of gene expression control under temperature stress is an essential step toward understanding the biochemical and physiological adaptation mechanism in invasive insect pests to cope with harsh conditions (Liu et al., 2017; Zhou et al., 2019). In this study, a detailed transcriptome analysis and characterization of the gene expression profiles of the FAW were evaluated under cold and high temperature stress, and transcriptome changes in larval stages were revealed through DEG analysis. Three transcriptomes were obtained from the larval stages of FAW exposed to various temperatures (4, 25, and 40°C) using RNA-seq techniques. In public databases (UniProt and NT), 14,338 (6.76%) and 33,699 (15.9%) unigenes were successfully annotated as minimum and maximum, respectively (Table 1). The findings are consistent with other transcriptome projects that have used Illumina technology (Zhu et al., 2012; Wu et al., 2015; Cui et al., 2017). The unigene sequences of 74.25% of the unigenes were most similar to gene sequences from *S. litura*, and there was a total of 96.2% similarity with the lepidopteran genus. This can be linked to the appropriate knowledge on lepidopteran transcriptomes and genomes. T40 had the most DEGs, with a wider distribution than T4 (Figures 2, 3), which is consistent with impact proteomics evidence from *Locusta migratoria* under high and low temperature stress (Ran et al., 2020).

Gene Ontology enrichment analysis enabled us to effectively identify critical biological processes associated with conditions of temperature stress (Li et al., 2020). According to GO analysis (Figure 4), the DEGs of FAW were significantly concentrated in the “metabolic mechanism,” “biological control,” “cell element,” “catalytic activity,” and “binding” under temperature stress. This indicates that when subjected to high- and low-temperature stress, the DEGs of the FAW often accumulate in pathways linked to energy metabolism, and it may suggest that the FAW primarily deals with temperature stress through energy metabolism and metabolic products. When insects are subjected to heat stress, the synthesis of the majority of proteins decreases (Li et al., 2020). Our DEG study has shown that many metabolic process genes have been suppressed at low and high temperatures (Figures 4A,B). The situation was similar in *Glyphodes pyloalis* (Liu et al., 2017) and *M. alternatus* (Li et al., 2020) exposed to 25 and 40°C. As a result, certain genes involved in the removal of defective proteins will be activated in order to preserve cellular structures and functions. Ubiquitin-mediated proteolysis (UMP) played an important role in cytoprotection by degrading damaged proteins (Plafker, 2010). After heat exposure, two ubiquitin-related unigenes of FAW (ubiquitin-conjugating enzyme E2 and von Hippel-Lindau disease tumor suppressor) were upregulated. The findings suggested that the UMP could play a role in the removal of damaged proteins during insect heat stress.

Kyoto Encyclopedia of Genes and Genomes analysis revealed that most of the cold-regulated DEGs (24 DEGs) were enriched in the “Metabolism” pathways’ including “Metabolic pathway,” “Carbon metabolism,” “Ubiquinone and other terpenoid-quinone biosynthesis,” “Purine metabolism,” “Biosynthesis of cofactors,” “Amino sugar and nucleotide sugar metabolism,” and “Biosynthesis of amino acids.” There are some similar results in the investigation on transcriptome responses to cold stress in the chrysomelidae, *G. daurica* (Zhou et al., 2019) and the ladybird, *C. montrouzieri* (Zhang et al., 2015a; Zhou et al., 2019) and also desert beetle *M. punctipennis* (Tusong et al., 2016). The concentration of KEGG under high temperature stress indicates the enrichment of various energy metabolism pathways in FAW under these conditions. The expression of fatty acid metabolism and unsaturated fatty acid synthesis pathways in its body is up-regulated in response to high temperatures, and a significant amount of fatty acid is stored to prevent water loss. A large number of amino acids are generated as a result of up-regulation of amino acid synthesis and metabolism, which provides raw materials for the synthesis of heat-resistant proteins (Ran et al., 2020). Under high temperature stress, FAW may start the degradation of macromolecular carbohydrates and produce small molecules of sugar, as well as accumulate and consume a variety of amino acids, as evidenced by the enrichment of a large number of energy metabolism pathways. These metabolic pathways can enhance the anti-reversibility of FAW for the resistance to high-temperature stress. The enrichment of “Protein processing in the endoplasmic reticulum” pathways in KEGG after heat exposure in *S. frugiperda* supported the hypothesis that the temperature accelerated protein unfolding and initiated molecular chaperones (Day et al., 2002). Transcriptome sequencing in *Bombyx mori* revealed that “longevity regulating pathway-multiple species” pathway was involved in diapause preparation (Chen et al., 2017), whereas this pathway contributed to heat tolerance in our research analysis that it is consistent with same study on a coleopteran insect, *M. alternatus* (Li et al., 2020). The enrichment of the “MAPK signaling pathway” in both stress conditions indicated that environmental stress could cause an FAW signal switch.

Differentially expresses gene enrichment revealed that unigenes involved in cellular processes, immune response, and signal transduction may be important components of the FAW heat-response mechanism (Figure 5B). Many immune response unigenes, such as apoptosis and lysozymes, were specifically induced in the high temperature treatment group, which were not observed in the low temperature treatment group. Furthermore, the balance between cell proliferation and apoptosis was critical for insect survival, and heat stress may trigger an increase in the incidence of apoptosis in organisms (Takayama et al., 2003; Cui et al., 2017). In practice, the insect immune system was the primary regulator of apoptosis. The enrichment of “lysozymes” was thought to play a role in the body’s protection against infection, while heat stress was thought to increase disease and shorten lifespan; thus, the induction of these genes confirmed that heat stress could elicit an immune response directly.

We also discovered two DEGs linked to odorant binding proteins (OBPs) in this study (Figure 6B and Supplementary Table 8), both of which (*OBP17* and *OBP2*5) were down-regulated at high temperatures. At low temperatures, *OPB25* was up-regulated. These findings suggest that at such high temperatures, chemosensory system of *S. frugiperda* is unable to react appropriately to chemical cues in the environment. Four *OBP*s were found to be upregulated and five were found to be downregulated in the third antennal segments of high-temperature-acclimated *Drosophila*. Temperature extremes and stress conditions such as starvation have both been linked to changes in certain *OBP*s (Jafari and Alenius, 2015).

A wide range of oxidative substances, including pesticides, plant secondary metabolites, and some oxidative substances catalyzed by cytochrome P450s (Daborn et al., 2007; Niu et al., 2011) and as a main enzyme, it could enrich “Antioxidant activity” pathway, and resist oxidative stress damage to the organism in low temperature (Zhou et al., 2019). We discovered that *cytochrome P450 CYP6B50* and *cytochrome P450 CYP306A1* up-regulated at low and high temperatures, respectively. Interestingly, another enzyme, *cytochrome P450 CYP4G75*, is up-regulated only at low temperatures and down-regulated only at high temperatures. Some cytochrome P450 genes were upregulated under both low and high temperatures in three rice plant hopper species in a comparative study of transcriptional responses to low and high temperatures, indicating that cold and heat stress increase oxidative stress in the insect body (Huang et al., 2017).

We found one cuticular protein gene (larval cuticle protein 1-like) that was upregulated from 119 DEGs after cold stress in this study (Figure 6B and Supplementary Table 8). Many other species have been shown to have cold-responsive cuticular protein genes such as flies (Qin et al., 2005), wasps (Colinet et al., 2007), beetles (Carrasco et al., 2011; Zhou et al., 2019), locusts (Wang et al., 2016), stick insects (Dunning et al., 2013), rice plant hoppers (Huang et al., 2017), and seabuckthorn carpenter moth (Cui et al., 2017), suggesting that the change in insect cuticle may play an important role in adaptation to low temperature.

## Conclusion

We used RNA-Seq technology focused on high-throughput sequencing to compare the transcriptomes of *S. frugiperda* under high- and low-temperature stresses. This research was the first to identify a large number of genes that were significantly up-regulated at high and low temperatures (Supplementary Tables 7, 8). Many genes were discovered through comparative transcriptome analysis, and a significant number of improvements in metabolic pathways were discovered through GO and KEGG enrichment analysis. Our findings will help future molecular research and genomic studies. These newly found genes may be important and necessary to FAW harsh environment tolerance and its behavior for adaptation in new environment as well as quarantine area.

## Data Availability Statement

NGS datasets were analyzed in this study has been deposited in the Gene Expression Omnibus (GEO) database (https://www.ncbi.nlm.nih.gov/geo/, accession number GSE175545) and the Sequence Read Archive (SRA) (https://www.ncbi.nlm.nih.gov/sra, accession number SRP321312).

## Author Contributions

YP conceived the idea and designed the experiments. MV and YP performed the experiments, analyzed the data, co-wrote the manuscript, discussed the results, and commented on the manuscript. Both authors contributed to the article and approved the submitted version.

## Conflict of Interest

The authors declare that the research was conducted in the absence of any commercial or financial relationships that could be construed as a potential conflict of interest.

## Publisher’s Note

All claims expressed in this article are solely those of the authors and do not necessarily represent those of their affiliated organizations, or those of the publisher, the editors and the reviewers. Any product that may be evaluated in this article, or claim that may be made by its manufacturer, is not guaranteed or endorsed by the publisher.

## Footnotes

1
http://www.usadellab.org/cms/?page=trimmomatic

2
http://www.bioinformatics.babraham.ac.uk/projects/fastqc/

3
http://weizhongli-lab.org/cd-hit

4
https://github.com/TransDecoder/TransDecoder/wiki

5
http://deweylab.github.io/RSEM/

6
http://www.genome.jp/kegg/ko.html

7
https://www.ncbi.nlm.nih.gov/nucleotide/

8
https://pfam.xfam.org/

9
http://www.geneontology.org/

10
https://www.ncbi.nlm.nih.gov/protein/

11
http://www.uniprot.org/

12
http://eggnogdb.embl.de/

13
https://github.com/bbuchfink/diamond

14
https://huygens.science.uva.nl/VolcaNoseR

15
http://www.heatmapper.ca/expression/

16
www.idtdna.com
