# Supplementary material for: Differential Transcriptome Analysis Reveals Genes Related to Low- and High-Temperature Stress in the Fall Armyworm, Spodoptera frugiperda
Source: Front Physiol. 2022 Jan 31;12:827077. doi: 10.3389/fphys.2021.827077 (PMC8841556; doi:10.3389/fphys.2021.827077)
Supplement: Supplementary file 1 [file Table_1.docx]

**Frontiers in Physiology**

**Differential transcriptome analysis reveals genes related to low- and high-temperature stress in the fall armyworm, *Spodoptera frugiperda***

**Mohammad Vatanparast and Youngjin Park^*^**

Plant Quarantine Technology center, Animal and Plant Quarantine Agency, Gimcheon, Republic of Korea

Running Title: Genes Related to Temperature Stress

^*^Corresponding author

Email) [parky1127@korea.kr](mailto:parky1127@korea.kr)

**Supporting Information**

**Supplementary Table S1.** **Primer sequences used in this study.**

Supplementary Table S1.

| Contig | Gene | Primer | Use | Orientation | Sequence (5ʹ - 3ʹ) | Annealing  temperature (°C) | Amplicon  (bp) |
| --- | --- | --- | --- | --- | --- | --- | --- |
| c214749_g1_i1 | venom serine protease 34-like isoform X1 | VSP_FAW_F | qPCR | Forward  Reverse | CCGTGATACATCCGTCGTTTAC  CCAACTGGCTGAACTCCATATT | 55 | 90 |
| c170666_g1_i1 | 3-ketodihydrosphingosine reductase | KDR_FAW | qPCR | Forward  Reverse | GCTTGTAGGCACAAATGTTAGG  TTGTCTCCTCTGGTTTGGTTAG | 53 | 98 |
| c180292_g9_i4 | SID1 transmembrane family member 2-like | SID_FAW | qPCR | Forward  Reverse | GCGTCCGTGGAGTTTATCTT  GTATCGCCACCGTCTGTTT | 52 | 92 |
| c170786_g1_i1 | phospholipase A1-like | PHA_FAW | qPCR | Forward  Reverse | ACGTGGATTTCTACCCGAATG  CACAGACTCGGCGTACAATAA | 52 | 95 |
| c179215_g2_i2 | juvenile hormone epoxide hydrolase-like | JHE_FAW | qPCR | Forward  Reverse | TCGATTACGGAGGCCATTTC GTTACTAGGTGGCGCCTTATT | 52 | 123 |
| c166591_g1_i1 | venom allergen 5.01-like | VAL_FAW | qPCR | Forward  Reverse | CCTCACCGAATCCGTCAAATA GGGATGGTGTTCGAGATGTAG | 53 | 108 |
| c182964_g5_i1 | proclotting enzyme-like | PCE_FAW | qPCR | Forward  Reverse | CACCCAAGTGTGGTTCTAACT  CTTGGTATGGGTAGGGTTCTTG | 54 | 112 |
| c180345_g2_i1 | heat shock protein 68-like | HSP_FAW | qPCR | Forward  Reverse | CGAGGAGTGCCTAAGATTGATG CGACCCTTGTCGTTCTTGATTA | 55 | 122 |
| c179097_g1_i1 | fatty acyl-CoA reductase wat-like | FAR_FAW | qPCR | Forward  Reverse | GCTCCAGTAGACATGGTGAATAA TGGAGCTGACGGTATAGATAGG | 54 | 103 |
| c185519_g3_i1 | filamin-A | FIL_FAW | qPCR | Forward  Reverse | GAAGTTGGTGATGCCAAGAAAG GTGTCTACGCTGAAGGTGTT | 54 | 89 |
|  | EF1α | Sf-EF1α | qPCR | Forward  Reverse | TGGGCGTCAACAAAATGGA  TCTCCGTGCCAGCCAGAAAT | 52 | 129 |
