# Supplementary material for: Differential Transcriptome Analysis Reveals Genes Related to Low- and High-Temperature Stress in the Fall Armyworm, Spodoptera frugiperda
Source: Front Physiol. 2022 Jan 31;12:827077. doi: 10.3389/fphys.2021.827077 (PMC8841556; doi:10.3389/fphys.2021.827077)
Supplement: Supplementary file 2 [file Table_2.docx]

**Frontiers in Physiology**

**Differential transcriptome analysis reveals genes related to low- and high-temperature stress in the fall armyworm, *Spodoptera frugiperda***

**Mohammad Vatanparast and Youngjin Park^*^**

Plant Quarantine Technology center, Animal and Plant Quarantine Agency, Gimcheon, Republic of Korea

Running Title: Genes Related to Temperature Stress

^*^Corresponding author

Email) [parky1127@korea.kr](mailto:parky1127@korea.kr)

**Supporting Information**

**Supplementary Table S2. Project information of transcriptome analysis.**

Supplementary Table S2.

| **Project information** | |
| --- | --- |
| Read Length | 101 |
| Library Kit | TruSeq Stranded mRNA LT Sample Prep Kit |
| Library Protocol | TruSeq Stranded mRNA Sample Preparation Guide, Part # 15031047 Rev. |
| Reagent | TruSeq 3000 4000 SBS Kit v3 |
| Sequencing Protocol | NovaSeq 6000 System User Guide Document #1000000019358 v02 |
| Sequencing Control Software | 1000000019358 v02 |
