# Supplementary material for: Differential Transcriptome Analysis Reveals Genes Related to Low- and High-Temperature Stress in the Fall Armyworm, Spodoptera frugiperda
Source: Front Physiol. 2022 Jan 31;12:827077. doi: 10.3389/fphys.2021.827077 (PMC8841556; doi:10.3389/fphys.2021.827077)
Supplement: Supplementary file 4 [file Table_4.docx]

**Frontiers in Physiology**

**Differential transcriptome analysis reveals genes related to low- and high-temperature stress in the fall armyworm, *Spodoptera frugiperda***

**Mohammad Vatanparast and Youngjin Park^*^**

Plant Quarantine Technology center, Animal and Plant Quarantine Agency, Gimcheon, Republic of Korea

Running Title: Genes Related to Temperature Stress

^*^Corresponding author

Email) [parky1127@korea.kr](mailto:parky1127@korea.kr)

**Supporting Information**

**Supplementary Table S4.** **Summary of data production.** Trimming data statistics results. Adapter sequences were removed. Q20 and Q30 are Ratio (%) of bases that have phred quality score greater than or equal to 20 or 30, respectively.

Supplementary Table S4.

| **Index** | **Sample ID** | **Total read bases** | **Total reads** | **GC (%)** | **Q20 (%)** | **Q30 (%)** |
| --- | --- | --- | --- | --- | --- | --- |
| 1 | T4 | 14,854,832,258 | 147,605,076 | 45.27 | 98.94 | 96.09 |
| 2 | T25 | 10,443,340,846 | 103,761,112 | 44.97 | 98.89 | 95.93 |
| 3 | T40 | 12,926,084,334 | 128,451,536 | 46.16 | 98.98 | 96.22 |
