# Supplementary material for: Differential Transcriptome Analysis Reveals Genes Related to Low- and High-Temperature Stress in the Fall Armyworm, Spodoptera frugiperda
Source: Front Physiol. 2022 Jan 31;12:827077. doi: 10.3389/fphys.2021.827077 (PMC8841556; doi:10.3389/fphys.2021.827077)
Supplement: Supplementary file 5 [file Table_5.docx]

**Frontiers in Physiology**

**Differential transcriptome analysis reveals genes related to low- and high-temperature stress in the fall armyworm, *Spodoptera frugiperda***

**Mohammad Vatanparast and Youngjin Park^*^**

Plant Quarantine Technology center, Animal and Plant Quarantine Agency, Gimcheon, Republic of Korea

Running Title: Genes Related to Temperature Stress

^*^Corresponding author

Email) [parky1127@korea.kr](mailto:parky1127@korea.kr)

**Supporting Information**

**Supplementary Table S5.** ***De novo* transcriptome assembly statistics.** The *de novo* assembly of merged data was carried out using Trinity software. N[x] length statistic: At least x% of the assembled transcript nucleotides are found in contigs that are at least of Nx length.

Supplementary Table S5.

| **Assembly** | **Merge** | | |
| --- | --- | --- | --- |
|  | **All transcript**  **contigs** | **Only longest**  **isoform per 'gene'** | |
| Total trinity 'genes' | 227,950 | | 227,950 |
| Total trinity transcripts | 318,058 | | 227,950 |
| Percent GC | 37.56 | | 36.91 |
| N90 | 279 | | 256 |
| N80 | 391 | | 335 |
| N70 | 535 | | 445 |
| N60 | 728 | | 593 |
| N50 | 1,021 | | 814 |
| N40 | 1,419 | | 1,176 |
| N30 | 1,964 | | 1,717 |
| N20 | 2,770 | | 2,515 |
| N10 | 4,263 | | 4,058 |
| Maximum contig length | 41,309 | | 41,309 |
| Minimum contig length | 201 | | 201 |
| Median contig length | 391.0 | | 349.0 |
| Average contig length | 685.18 | | 602.59 |
| Total assembled bases | 217,925,485 | | 137,360,884 |
