# Supplementary material for: Differential Transcriptome Analysis Reveals Genes Related to Low- and High-Temperature Stress in the Fall Armyworm, Spodoptera frugiperda
Source: Front Physiol. 2022 Jan 31;12:827077. doi: 10.3389/fphys.2021.827077 (PMC8841556; doi:10.3389/fphys.2021.827077)
Supplement: Supplementary file 6 [file Table_6.docx]

**Frontiers in Physiology**

**Differential transcriptome analysis reveals genes related to low- and high-temperature stress in the fall armyworm, *Spodoptera frugiperda***

**Mohammad Vatanparast and Youngjin Park^*^**

Plant Quarantine Technology center, Animal and Plant Quarantine Agency, Gimcheon, Republic of Korea

Running Title: Genes Related to Temperature Stress

^*^Corresponding author

Email) [parky1127@korea.kr](mailto:parky1127@korea.kr)

**Supporting Information**

**Supplementary Table S6.** **Clustering transcripts into unigenes.** Longest contigs of the assembled contigs are filtered and clustered into the non-redundant transcripts using CD-HIT-EST program. N[x] length statistic: At least x% of the assembled transcript nucleotides are found in contigs that are at least of Nx length.

Supplementary Table S6.

| **Assembly** | **Merge** | |
| --- | --- | --- |
|  | **Only longest isoform**  **per 'gene'** | **Clustered Contig**  **‘Unigene’** |
| Total 'genes' | 227,950 | 211,967 |
| Percent GC | 36.91 | 36.75 |
| N90 | 256 | 260 |
| N80 | 335 | 345 |
| N70 | 445 | 461 |
| N60 | 593 | 617 |
| N50 | 814 | 853 |
| N40 | 1,176 | 1,229 |
| N30 | 1,717 | 1,782 |
| N20 | 2,515 | 2,586 |
| N10 | 4,058 | 4,140 |
| Maximum contig length | 41,309 | 41,309 |
| Minimum contig length | 201 | 201 |
| Median contig length | 349.0 | 356.0 |
| Average contig length | 602.59 | 618.67 |
| Total assembled bases | 137,360,884 | 131,138,474 |
