# Supplementary material for: Differential Transcriptome Analysis Reveals Genes Related to Low- and High-Temperature Stress in the Fall Armyworm, Spodoptera frugiperda
Source: Front Physiol. 2022 Jan 31;12:827077. doi: 10.3389/fphys.2021.827077 (PMC8841556; doi:10.3389/fphys.2021.827077)
Supplement: Supplementary file 7 [file Table_7.docx]

**Frontiers in Physiology**

**Differential transcriptome analysis reveals genes related to low- and high-temperature stress in the fall armyworm, *Spodoptera frugiperda***

**Mohammad Vatanparast and Youngjin Park^*^**

Plant Quarantine Technology center, Animal and Plant Quarantine Agency, Gimcheon, Republic of Korea

Running Title: Genes Related to Temperature Stress

^*^Corresponding author

Email) [parky1127@korea.kr](mailto:parky1127@korea.kr)

**Supporting Information**

**Supplementary Table S7.** **Associated genes related to pathways enrichment base on KEGG classification (*P*-value<0.01, log_2_FC≥5, ≤-5).**

Supplementary Table S7.

| **Query ID** | **Unigene** | ***P*- value** | **Target length** | **Bit** | ***E*-value** | **Identities (%)** | **KEGG number** | **Enriched KEGG pathways** |
| --- | --- | --- | --- | --- | --- | --- | --- | --- |
| **T4/T25** | | | | | | | | |
| c185718_g1_i2 | Replication factor C subunit 2 | 0.008347364 | 1455 | 593.9 | 2E-165 | 88 | haw:110380397 | DNA replication |
|  |  |  |  |  |  |  |  | Nucleotide excision repair |
|  |  |  |  |  |  |  |  | Mismatch repair |
| c179501_g3_i10 | Low quality protein: optomotor-blind protein | 0.009931865 | 3106 | 2614.1 | 0 | 98 | prap:110993331 | MAPK signaling pathway |
| c181038_g5_i1 | Glucose-6-phosphate isomerase | 0.004442385 | 2239 | 2423.9 | 0 | 90 | tnl:113494316 | Metabolic pathway |
|  |  |  |  |  |  |  |  | Starch and sucrose metabolism |
|  |  |  |  |  |  |  |  | Glycolysis/Gluconeogenesis |
|  |  |  |  |  |  |  |  | Pentose phosphate pathway |
|  |  |  |  |  |  |  |  | Carbon metabolism |
|  |  |  |  |  |  |  |  | Amino sugar and nucleotide sugar metabolism |
| c172758_g1_i1 | Adenylate kinase isoenzyme 1-like isoform X1 | 0.00676631 | 802 | 1186.7 | 0 | 93 | haw:110379686 | Metabolic pathway  Biosynthesis of cofactors  Purine metabolism  Thiamine metabolism |
| c181084_g1_i1 | 4-coumarate--coa ligase 1-like | 0.006181384 | 2403 | 2494.1 | 0 | 88 | haw:110384553 | Ubiquinone and other terpenoid-quinone biosynthesis |
| c181716_g9_i2 | Uracil phosphoribosyltransferase homolog | 0.008235804 | 1431 | 1160.8 | 0 | 84 | haw:110374559 | Pyrimidine metabolism |
| c176435_g4_i1 | Trypsin, alkaline C-like isoform X1 | 0.004087239 | 1425 | 935.2 | 0 | 95 | haw:110383548 | Neuroactive ligand-receptor interaction |
| c176700_g1_i1 | D-3-phosphoglycerate dehydrogenase | 0.000326504 | 1935 | 1417.5 | 0 | 90 | haw:110372452 | Glycine, serine and threonine metabolism |
|  |  |  |  |  |  |  |  | Cysteine and methionine metabolism |
|  |  |  |  |  |  |  |  | Metabolic pathways |
|  |  |  |  |  |  |  |  | Carbon metabolism |
|  |  |  |  |  |  |  |  | Biosynthesis of amino acids |
| c185128_g3_i2 | 4-coumarate--coa ligase 1-like | 0.000220491 | 2018 | 2451.6 | 0 | 90 | haw:110375840 | Metabolic pathways  Ubiquinone and other terpenoid-quinone biosynthesis |
| c136924_g1_i1 | Lutropin-choriogonadotropic hormone receptor | 0.028381911 | 3183 | 1194.1 | 0 | 94 | haw:110375110 | Wnt signaling pathway |
| **T40/t25** | | | | | | | | |
| c172209_g1_i1 | Histone deacetylase complex subunit SAP18 | 0.002022 | 174 | 352.1 | 2E-93 | 97 | bmor:101739989 | RNA transport  mRNA surveillance pathway |
| c172374_g13_i3 | Camp-specific 3',5'-cyclic phosphodiesterase | 3.96E-05 | 531 | 1060.8 | 2E-306 | 95 | bmor:101742254 | Purine metabolism  Metabolic pathways |
| c171850_g1_i1 | Small nuclear ribonucleoprotein Sm D3 | 0.003932 |  |  |  |  | bmor:101744663 | Spliceosome |
| c177350_g2_i1 | Putative RDH13 | 0.00112 | 169 | 144.8 | 3E-31 | 59 | dpl:KGM_206844 | Retinol metabolism  Metabolic pathways  Biosynthesis of cofactors |
| c185159_g1_i4 | Putative Vacuolar ATP synthase subunit C | 0.003747 | 195 | 403.7 | 6E-109 | 98 | dpl:KGM_215153 | Oxidative phosphorylation  Metabolic pathways  Phagosome  mTOR signaling pathway |
| c172207_g1_i1 | Peroxisomal membrane protein 2 | 0.000886 | 220 | 425.2 | 2E-115 | 94 | haw:110369735 | Galactose metabolism  Starch and sucrose metabolism  Metabolic pathways  Lysosome |
| c187476_g4_i1 | Ca(2+)/calmodulin-responsive adenylate cyclase-like | 0.000272 | 462 | 511.1 | 3E-141 | 99 | haw:110369899 | Purine metabolism  Metabolic pathways  Longevity regulating pathway - multiple species |
| c182201_g1_i2 | Probable Golgi SNAP receptor complex member 2 | 0.005222 | 553 | 113.2 | 2E-21 | 100 | haw:110369989 | SNARE interactions in vesicular transport |
| c164082_g1_i2 | Mpv17-like protein | 0.003264 | 260 | 58.9 | 0.00007 | 93 | haw:110370006 | Peroxisome |
| c181300_g1_i2 | Glycerol-3-phosphate phosphatase-like | 0.004426 |  |  |  |  | haw:110370042 | Glyoxylate and dicarboxylate metabolism  Metabolic pathways  Carbon metabolism |
| c182334_g1_i1 | Histone-lysine N-methyltransferase SUV39H2-like isoform X1 | 0.002348 | 286 | 417.5 | 4E-113 | 95 | haw:110370326 | Lysine degradation  Metabolic pathways |
| c188689_g3_i1 | UDP-glucose:glycoprotein glucosyltransferase | 0.0004 | 130 | 223.8 | 9E-55 | 100 | haw:110370674 | Protein processing in endoplasmic reticulum |
| c185848_g1_i1 | Xanthine dehydrogenase | 0.001362 | 185 | 349 | 3E-92 | 98 | haw:110370678 | Pyrimidine metabolism  Metabolic pathways |
| c183309_g1_i1 | Glucose dehydrogenase [FAD, quinone]-like | 0.004855 | 159 | 330.5 | 5E-87 | 99 | haw:110370921 | Pentose phosphate pathway  Metabolic pathways |
| c179237_g2_i1 | Peptidoglycan-recognition protein LB-like | 0.008911 | 275 | 558.5 | 1E-155 | 92 | haw:110370939 | Toll and Imd signaling pathway |
| c188466_g7_i1 | Dolichyl pyrophosphate Man9GlcNAc2 alpha-1,3-glucosyltransferase | 0.002094 | 750 | 237.3 | 1E-58 | 98 | haw:110370956 | N-Glycan biosynthesis  Metabolic pathways |
| c185767_g1_i3 | Replication factor C subunit 4 | 0.003579 | 294 | 307 | 5E-80 | 91 | haw:110371094 | DNA replication  Nucleotide excision repair  Mismatch repair |
| c178086_g2_i2 | Glutathione S-transferase 1-like | 0.000163 | 425 | 55.8 | 0.0005 | 92 | haw:110371344 | Glutathione metabolism  Metabolism of xenobiotics by cytochrome P450  Drug metabolism - cytochrome P450  Drug metabolism - other enzymes  Metabolic pathways |
| c166805_g1_i1 | Eukaryotic translation initiation factor 3 subunit D | 0.000193 | 300 | 617.1 | 5E-173 | 98 | haw:110371347 | RNA transport |
| c189621_g1_i1 | Alpha-L-fucosidase isoform X1 | 0.000949 | 951 | 528.1 | 2E-146 | 92 | haw:110371508 | Other glycan degradation  Lysosome |
| c183175_g5_i2 | UDP-N-acetylglucosamine--dolichyl-phosphate N-acetylglucosaminephosphotransferase | 0.002583 | 214 | 416.8 | 8E-113 | 96 | haw:110371586 | N-Glycan biosynthesis  Metabolic pathways |
| c186749_g1_i1 | Histidine--trna ligase, cytoplasmic isoform X1 | 0.002525 |  |  |  |  | haw:110371792 | Aminoacyl-tRNA biosynthesis |
| c178155_g1_i1 | Adenylosuccinate lyase | 0.00311 | 495 | 813.9 | 3E-232 | 97 | haw:110372045 | Purine metabolism  Alanine, aspartate and glutamate metabolism  Metabolic pathways  Biosynthesis of cofactors |
| c175787_g1_i2 | Endonuclease III-like protein 1 | 0.001864 | 500 | 654.8 | 2E-184 | 90 | haw:110372064 | Base excision repair |
| c176977_g1_i1 | Uroporphyrinogen decarboxylase | 0.000563 | 429 | 856.3 | 7E-245 | 99 | haw:110372329 | Porphyrin and chlorophyll metabolism  Metabolic pathways  Biosynthesis of cofactors |
| c181921_g4_i2 | L-lactate dehydrogenase-like isoform X1 | 0.002755 | 539 | 1057.4 | 2E-305 | 96 | haw:110372435 | Glycolysis / Gluconeogenesis  Cysteine and methionine metabolism  Pyruvate metabolism  Propanoate metabolism  Metabolic pathways |
| c166854_g2_i1 | Arginine kinase isoform X1 | 0.007333 | 569 | 1119.4 | 0 | 99 | haw:110372888 | Arginine and proline metabolism |
| c185583_g1_i7 | DNA-directed RNA polymerase III subunit RPC3 | 0.001075 | 363 | 719.2 | 1E-203 | 98 | haw:110373286 | RNA polymerase |
| c175843_g1_i1 | Phosphatidylinositol glycan anchor biosynthesis class U protein | 0.002146 | 182 | 169.1 | 2E-38 | 69 | haw:110374083 | Glycosylphosphatidylinositol(GPI)-anchor biosynthesis  Metabolic pathways |
| c181456_g3_i1 | Vesicle-trafficking protein SEC22b | 0.00035 |  |  |  |  | haw:110374665 | SNARE interactions in vesicular transport  Phagosome |
| c188293_g5_i1 | Folliculin-interacting protein 2 isoform X1 | 0.000232 | 483 | 963.4 | 5E-277 | 98 | haw:110374815 | mTOR signaling pathway |
| c173799_g1_i1 | Probable very-long-chain enoyl-coa reductase art-1 isoform X1 | 9.09E-05 | 420 | 828.2 | 2E-236 | 99 | haw:110375119 | Fatty acid elongation  Biosynthesis of unsaturated fatty acids  Metabolic pathways  Fatty acid metabolism |
| c182700_g1_i1 | Sphingomyelin phosphodiesterase isoform X1 | 3.74E-05 | 707 | 1424.5 | 0 | 99 | haw:110375162 | Sphingolipid metabolism  Metabolic pathways  Lysosome |
| c187487_g4_i5 | 1-acyl-sn-glycerol-3-phosphate acyltransferase gamma-like | 0.000118 | 628 | 980.3 | 4E-282 | 82 | haw:110375219 | Glycerolipid metabolism  Glycerophospholipid metabolism  Metabolic pathways |
| c188350_g2_i2 | PR domain zinc finger protein 16-like | 0.004262 | 543 | 71.6 | 4E-09 | 89 | haw:110375373 | Lysine degradation  Metabolic pathways |
| c184792_g1_i2 | GPI inositol-deacylase | 0.000549 | 230 | 424.1 | 4E-115 | 90 | haw:110375401 | Glycosylphosphatidylinositol (GPI)-anchor biosynthesis  Metabolic pathways |
| c110176_g1_i1 | ATP synthase subunit d, mitochondrial | 0.002032 | 75 | 120.2 | 5E-23 | 93 | haw:110375576 | Oxidative phosphorylation  Metabolic pathways |
| c181703_g1_i5 | Protein msta-like isoform X1 | 0.00059 | 234 | 477.2 | 5E-131 | 93 | haw:110375831 | Other glycan degradation  Lysosome |
| c189049_g2_i3 | Translocator protein-like isoform X1 | 0.002175 | 463 | 829.3 | 1E-236 | 87 | haw:110376024 | Neuroactive ligand-receptor interaction  Apoptosis - fly |
| c185580_g1_i1 | Aminopeptidase N-like isoform X1 | 0.007018 | 291 | 588.2 | 2E-164 | 98 | haw:110376037 | Glutathione metabolism  Metabolic pathways |
| c177570_g6_i1 | Dolichyl-diphosphooligosaccharide--protein glycosyltransferase subunit STT3A | 0.001744 | 419 | 400.2 | 1E-107 | 99 | haw:110376573 | N-Glycan biosynthesis  Various types of N-glycan biosynthesis  Metabolic pathways  Protein processing in endoplasmic reticulum |
| c186904_g1_i3 | CDP-diacylglycerol--glycerol-3-phosphate 3-phosphatidyltransferase, mitochondrial | 0.000496 | 191 | 383.3 | 1E-102 | 93 | haw:110376728 | Glycerophospholipid metabolism  Metabolic pathways |
| c179318_g1_i1 | Chitinase-3-like protein 2 | 0.001125 | 335 | 646 | 1E-181 | 96 | haw:110376874 | Amino sugar and nucleotide sugar metabolism  Metabolic pathways |
| c175237_g1_i1 | Small nuclear ribonucleoprotein-associated protein B | 2.1E-05 | 217 | 433.7 | 1E-117 | 100 | haw:110376929 | Spliceosome |
| c181777_g1_i1 | Mannose-1-phosphate guanyltransferase beta | 0.000191 | 282 | 578.6 | 2E-161 | 98 | haw:110377210 | Fructose and mannose metabolism  Amino sugar and nucleotide sugar metabolism  Metabolic pathways  Biosynthesis of cofactors |
| c180609_g1_i3 | Lipoyltransferase 1, mitochondrial isoform X1 | 0.002127 | 520 | 1036.9 | 2E-299 | 96 | haw:110377750 | Lipoic acid metabolism  Metabolic pathways  Biosynthesis of cofactors |
| c182088_g1_i2 | Aminopeptidase N-like | 0.002029 | 369 | 689.5 | 1E-194 | 99 | haw:110378026 | Glutathione metabolism  Metabolic pathways |
| c187580_g2_i2 | TFIIH basal transcription factor complex helicase XPD subunit | 0.003648 | 363 | 737.3 | 4E-209 | 96 | haw:110378851 | Basal transcription factors  Nucleotide excision repair |
| c188297_g1_i1 | Ubiquitin-conjugating enzyme E2-22 kda | 0.001407 | 347 | 605.9 | 5E-169 | 90 | haw:110378888 | Ubiquitin mediated proteolysis |
| c177547_g1_i1 | Lipase 1-like | 0.002391 | 974 | 1650.2 | 0 | 86 | haw:110379095 | Steroid biosynthesis  Lysosome |
| c188144_g1_i1 | TBC1 domain family member 15 isoform X1 | 0.003425 | 209 | 407.1 | 1E-109 | 98 | haw:110379129 | Mitophagy - animal |
| c185091_g2_i1 | 26S protease regulatory subunit 6A-B | 0.005285 | 593 | 1000.7 | 2E-288 | 92 | haw:110379244 | Proteasome |
| c163389_g1_i2 | Von Hippel-Lindau disease tumor suppressor | 0.001649 |  |  |  |  | haw:110379515 | Ubiquitin mediated proteolysis |
| c187076_g3_i1 | Peroxisomal membrane protein PMP34 | 0.00013 | 692 | 1465.3 | 0 | 99 | haw:110379619 | Peroxisome |
| c184208_g1_i1 | Adenosine kinase-like | 0.000261 |  |  |  |  | haw:110379865 | Purine metabolism  Metabolic pathways |
| c184496_g1_i1 | Nucleoporin Nup43 | 0.000257 | 213 | 415.6 | 4E-112 | 93 | haw:110380764 | RNA transport |
| c86983_g1_i1 | Caltractin-like isoform X1 | 0.000315 | 395 | 462.6 | 2E-126 | 93 | haw:110380819 | Nucleotide excision repair |
| c186014_g1_i3 | CDK-activating kinase assembly factor MAT1 | 0.006813 | 824 | 999.6 | 7E-288 | 95 | haw:110381013 | Basal transcription factors  Nucleotide excision repair |
| c182793_g1_i1 | UMP-CMP kinase | 0.007297 | 398 | 796.2 | 6E-227 | 97 | haw:110381293 | Pyrimidine metabolism  Drug metabolism - other enzymes  Metabolic pathways  Biosynthesis of cofactors |
| c185600_g1_i2 | GPI mannosyltransferase 3 | 0.001397 | 357 | 674.1 | 8E-190 | 92 | haw:110381785 | Glycosylphosphatidylinositol (GPI)-anchor biosynthesis  Metabolic pathways |
| c177932_g1_i1 | Dolichyl-diphosphooligosaccharide--protein glycosyltransferase subunit 2 | 0.002127 | 346 | 649.8 | 7E-183 | 99 | haw:110381826 | N-Glycan biosynthesis  Various types of N-glycan biosynthesis  Metabolic pathways  Protein processing in endoplasmic reticulum |
| c172254_g2_i2 | Structure-specific endonuclease subunit SLX1 homolog | 0.007382 | 1024 | 1414.4 | 0 | 75 | haw:110381984 | Fanconi anemia pathway |
| c177515_g8_i1 | 6-phosphogluconate dehydrogenase, decarboxylating | 0.00188 |  |  |  |  | haw:110382514 | Pentose phosphate pathway  Glutathione metabolism  Metabolic pathways  Carbon metabolism |
| c185612_g1_i1 | UDP-glucuronosyltransferase 2B31-like | 0.002747 |  |  |  |  | haw:110382758 | Pentose and glucuronate interconversions  Ascorbate and aldarate metabolism  Retinol metabolism  Porphyrin and chlorophyll metabolism  Metabolism of xenobiotics by cytochrome P450  Drug metabolism - cytochrome P450  Drug metabolism - other enzymes  Metabolic pathways  Biosynthesis of cofactors |
| c188102_g1_i1 | Pre-mrna-processing-splicing factor 8 isoform X1 | 0.002123 | 147 | 231.5 | 2E-57 | 99 | haw:110383687 | Spliceosome |
| c181779_g1_i1 | Beta-1,3-galactosyltransferase 6 | 0.006094 | 966 | 1817.4 | 0 | 92 | haw:110383736 | Glycosaminoglycan biosynthesis - chondroitin sulfate / dermatan sulfate  Glycosaminoglycan biosynthesis - heparan sulfate / heparin  Metabolic pathways |
| c174669_g1_i2 | Glutathione S-transferase 1-like | 0.002231 | 507 | 972.2 | 8E-280 | 95 | haw:110383926 | Glutathione metabolism  Metabolism of xenobiotics by cytochrome P450  Drug metabolism - cytochrome P450  Drug metabolism - other enzymes  Metabolic pathways |
| c175890_g1_i1 | Mannosyl-oligosaccharide 1,2-alpha-mannosidase IA | 0.001811 | 514 | 1011.1 | 2E-291 | 96 | haw:110383953 | N-Glycan biosynthesis  Various types of N-glycan biosynthesis  Metabolic pathways  Protein processing in endoplasmic reticulum |
| c173048_g1_i1 | DNA excision repair protein ERCC-1 | 0.000481 | 515 | 797 | 3E-227 | 86 | haw:110383957 | Nucleotide excision repair  Fanconi anemia pathway |
| c144730_g1_i1 | Non-homologous end-joining factor 1-like | 0.00287 | 662 | 1188.7 | 0 | 99 | haw:110383963 | Non-homologous end-joining |
| c188804_g3_i1 | Lysosomal alpha-glucosidase-like | 0.003774 | 927 | 1905.6 | 0 | 99 | haw:110384254 | Galactose metabolism  Starch and sucrose metabolism  Metabolic pathways  Lysosome |
| c177225_g1_i2 | Deoxycytidylate deaminase | 0.002306 | 351 | 686.4 | 6E-194 | 98 | pbar:105432019 | Pyrimidine metabolism  Metabolic pathways |
| c182567_g6_i1 | Dual specificity mitogen-activated protein kinase kinase dsor1 | 0.0002 | 1364 | 1434.5 | 0 | 97 | pmac:106716354 | MAPK signaling pathway - fly  FoxO signaling pathway  Autophagy - animal  mTOR signaling pathway  Dorso-ventral axis formation |
| c120060_g1_i1 | Diacylglycerol kinase epsilon | 0.006094 | 309 | 614.8 | 3E-172 | 98 | tnl:113491976 | Glycerolipid metabolism  Glycerophospholipid metabolism  Metabolic pathways  Phosphatidylinositol signaling system |
| c165790_g2_i2 | Elongation factor 1-alpha 2 | 0.000174 | 523 | 1026.2 | 6E-296 | 98 | tnl:113492739 | RNA transport |
| c175646_g1_i1 | Lethal(2)neighbour of tid protein 2 | 0.002391 | 465 | 913.3 | 4E-262 | 95 | tnl:113492747 | N-Glycan biosynthesis  Various types of N-glycan biosynthesis  Metabolic pathways |
| c176000_g1_i2 | Serine/threonine-protein kinase polo isoform X1 | 0.004329 | 279 | 491.5 | 5E-135 | 99 | tnl:113492842 | FoxO signaling pathway |
| c185262_g5_i1 | Large neutral amino acids transporter small subunit 2 | 0.001376 | 688 | 904.4 | 2E-259 | 91 | tnl:113493326 | mTOR signaling pathway |
| c165416_g1_i1 | 26S proteasome non-atpase regulatory subunit 9 | 0.004482 | 963 | 1891.3 | 0 | 97 | tnl:113494135 | Proteasome |
| c179657_g1_i1 | N-acetylglucosaminyl-phosphatidylinositol de-N-acetylase | 0.003668 | 1630 | 100.1 | 1E-17 | 100 | tnl:113494428 | Glycosylphosphatidylinositol (GPI)-anchor biosynthesis  Metabolic pathways |
| c187166_g1_i3 | Translation initiation factor eif-2B subunit delta isoform X1 | 0.008393 | 383 | 786.6 | 2E-223 | 98 | tnl:113496780 | RNA transport |
| c182786_g1_i2 | Insulin receptor substrate 2-B isoform X1 | 0.001265 | 760 | 1474.1 | 0 | 97 | tnl:113497656 | FoxO signaling pathway  Autophagy - animal  mTOR signaling pathway  Longevity regulating pathway - multiple species |
| c188494_g1_i2 | Adenylate cyclase type 2-like isoform X1 | 0.000323 | 2364 | 4778.4 | 0 | 99 | tnl:113497935 | Purine metabolism  Metabolic pathways  Longevity regulating pathway - multiple species |
| c187292_g1_i3 | Biotin--protein ligase | 0.003083 | 654 | 1286.6 | 0 | 96 | tnl:113499624 | Biotin metabolism  Metabolic pathways |
| c180814_g1_i4 | Peptidoglycan recognition protein | 0.001043 | 1055 | 1328.9 | 0 | 97 | tnl:113500602 | Toll and Imd signaling pathway |
| c189726_g1_i1 | Collagen alpha-1(IV) chain | 0.000472 | 199 | 401.4 | 1E-107 | 99 | tnl:113501211 | ECM-receptor interaction |
| c185621_g3_i3 | Cytoplasmic dynein 1 intermediate chain isoform X1 | 0.000722 | 965 | 1630.9 | 0 | 98 | tnl:113501446 | Phagosome |
| c183997_g2_i3 | DNA primase small subunit | 0.001647 | 538 | 1068.1 | 0 | 97 | tnl:113504498 | DNA replication |
| c188568_g1_i2 | Protein sel-1 homolog 1 isoform X1 | 4.51E-05 | 254 | 444.1 | 6E-121 | 95 | tnl:113504919 | Protein processing in endoplasmic reticulum |
| c173729_g1_i1 | 28S ribosomal protein S5, mitochondrial-like | 0.000904 | 740 | 968.8 | 1E-278 | 97 | tnl:113504935 | Ribosome |
| c185638_g7_i1 | Neutral alpha-glucosidase AB-like isoform X1 | 0.000367 | 1570 | 3016.1 | 0 | 97 | tnl:113505102 | N-Glycan biosynthesis  Metabolic pathways  Protein processing in endoplasmic reticulum |
| c181691_g1_i2 | Phospholipid phosphatase 5-like | 0.00052 | 971 | 1598.9 | 0 | 78 | tnl:113505638 | Glycerolipid metabolism  Glycerophospholipid metabolism |
| c177994_g10_i1 | Cytosol aminopeptidase-like | 0.001176 | 186 | 378.6 | 4E-101 | 93 | tnl:113505796 | Arginine and proline metabolism  Glutathione metabolism  Metabolic pathways |
| c171520_g2_i1 | Dehydrodolichyl diphosphate synthase complex subunit DHDDS | 4.29E-05 | 486 | 924.5 | 2E-265 | 93 | tnl:113507305 | Terpenoid backbone biosynthesis |
| c173858_g1_i2 | Soluble guanylate cyclase 88E-like | 0.002079 | 1801 | 592 | 7E-165 | 98 | tnl:113507785 | Purine metabolism  Metabolic pathways |
| c169290_g2_i2 | Acetyl-coa carboxylase | 0.000472 | 189 | 280 | 7E-72 | 87 | bmor:101738903 | Fatty acid biosynthesis  Pyruvate metabolism  Propanoate metabolism  Metabolic pathways  Fatty acid metabolism |
