# Supplementary material for: Differential Transcriptome Analysis Reveals Genes Related to Low- and High-Temperature Stress in the Fall Armyworm, Spodoptera frugiperda
Source: Front Physiol. 2022 Jan 31;12:827077. doi: 10.3389/fphys.2021.827077 (PMC8841556; doi:10.3389/fphys.2021.827077)
Supplement: Supplementary file 8 [file Table_8.docx]

**Frontiers in Physiology**

**Differential transcriptome analysis reveals genes related to low- and high-temperature stress in the fall armyworm, *Spodoptera frugiperda***

**Mohammad Vatanparast and Youngjin Park^*^**

Plant Quarantine Technology center, Animal and Plant Quarantine Agency, Gimcheon, Republic of Korea

Running Title: Genes Related to Temperature Stress

^*^Corresponding author

Email) [parky1127@korea.kr](mailto:parky1127@korea.kr)

**Supporting Information**

**Supplementary Table S8.** **Same DEGs between T4 and T40 in comparison with T25 related to Heatmaps (*P*-value<0.01, log_2_FC≥5, ≤-5).**

Supplementary Table S8.

| **No** | **Contig** | **Gene** | **Gene bank accession number** | ***P*-value** | **Target length** | **Bit** | ***E*-value** | **Identities (%)** | **Fold change** | | **FPKM** | |
| --- | --- | --- | --- | --- | --- | --- | --- | --- | --- | --- | --- | --- |
|  |  |  |  |  |  |  |  |  | **T4/T25** | **T40/T25** | **T4/T25** | **T40/T25** |
| 1 | c133356_g1_i1 | Mg7 | AFI57045.1 | 7.65E-27 | 322 | 534.6 | 4E-148 | 94 | 22.630438 | 6010.106959 | 22.07692 | 6753.308 |
| 2 | c148378_g1_i1 | Cholinesterase 1-like | XP_022829783.1 | 7.53E-20 | 143 | 222.2 | 2E-54 | 73 | 67.980678 | 1967.579838 | 76 | 2540 |
| 3 | c183854_g3_i1 | Esterase FE4-like | XP_022818112.1 | 1.53E-21 | 1757 | 2078.6 | 0 | 88 | 116.587962 | 1783.677524 | 129.25 | 2284.25 |
| 4 | c155555_g4_i1 | Pancreatic triacylglycerol lipase-like | XP_022824778.1 | 7.27E-19 | 672 | 1290.8 | 0 | 94 | 191.970554 | 1558.862598 | 189.8571 | 1773 |
| 5 | c180402_g1_i1 | Alpha-(1,3)-fucosyltransferase C-like | XP_022831169.1 | 1.96E-20 | 590 | 137.1 | 3E-29 | 80 | 15.711086 | 1383.833605 | 16.66667 | 1708 |
| 6 | c183114_g2_i3 | L-dopachrome tautomerase yellow-f2-like | XP_022831247.1 | 2.53E-16 | 686 | 176.8 | 4E-41 | 92 | 22.956421 | 858.204471 | 19 | 819.5 |
| 7 | c179826_g3_i2 | Peritrophin-like protein | AGC94490.1 | 4.94E-22 | 122 | 241.1 | 2E-60 | 95 | 93.861638 | 736.076094 | 88.71084 | 803.7229 |
| 8 | c178630_g4_i1 | Chlorophyllide A binding protein | bmor:692624 | 1.53E-21 | 554 | 1061.6 | 9E-307 | 93 | 44.927736 | 592.553803 | 42.27419 | 645.5806 |
| 9 | c179215_g2_i2 | Juvenile hormone epoxide hydrolase-like | XP_022828204.1 | 3.46E-20 | 134 | 259.2 | 3E-65 | 92 | 7.682506 | 556.870141 | 7.529412 | 630.2353 |
| 10 | c147654_g1_i1 | Cytochrome b5-like | XP_022823555.1 | 8.35E-14 | 63 | 124.4 | 4E-25 | 87 | 31.961273 | 475.073061 | 32 | 544.9333 |
| 11 | c189635_g1_i1 | Cytochrome P450 CYP6B50 | AGO62004.1 | 3.57E-20 | 190 | 362.8 | 4E-96 | 99 | 90.914980 | 470.784083 | 85.75 | 514.1563 |
| 12 | c179272_g1_i2 | Monocarboxylate transporter 9-like | XP_022821635.1 | 8.46E-20 | 197 | 354 | 8E-94 | 97 | 15.401491 | 442.959425 | 14.41935 | 480.4194 |
| 13 | c181544_g1_i1 | Juvenile hormone esterase-like | XP_022827863.1 | 2E-20 | 335 | 409.1 | 1E-110 | 85 | 13.461762 | 409.759760 | 12.6768 | 446.656 |
| 14 | c179089_g6_i2 | Phosphoglycolate phosphatase 1B, chloroplastic-like | XP_022827987.1 | 1.79E-17 | 334 | 441 | 2E-120 | 93 | 66.761197 | 383.025313 | 61.81818 | 410.5455 |
| 15 | c178259_g1_i1 | Bile salt-activated lipase-like | XP_022822371.1 | 1.91E-18 | 3735 | 7041.8 | 0 | 92 | 7.890203 | 359.591139 | 7.4 | 391.65 |
| 16 | c169747_g2_i1 | Trypsin delta-like | XP_022825250.1 | 3.14E-14 | 234 | 467.6 | 3E-128 | 95 | 33.919908 | 317.583155 | 33.5 | 361.8 |
| 17 | c177682_g4_i1 | Antennal esterase CXE14 | ACV60241.1 | 9.61E-19 | 590 | 412.5 | 9E-112 | 87 | 18.471506 | 316.743997 | 17.32653 | 343.8163 |
| 18 | c215129_g1_i1 | Esterase E4-like | XP_022817164.1 | 7.67E-18 | 2026 | 2412.8 | 0 | 88 | 8.008573 | 297.655640 | 7.555556 | 325.8889 |
| 19 | c178547_g3_i1 | Lactase-phlorizin hydrolase-like | XP_022827201.1 | 2.65E-16 | 141 | 166.8 | 1E-37 | 78 | 8.691628 | 289.173885 | 8.142857 | 312.7143 |
| 20 | c148792_g1_i1 | Fatty acid-binding protein 1-like | XP_022831663.1 | 8.58E-19 | 264 | 545.4 | 1E-151 | 96 | 5.266389 | 278.007906 | 4.961255 | 303.1181 |
| 21 | c179987_g1_i1 | Nose resistant to fluoxetine protein 6-like | XP_022814683.1 | 3.83E-18 | 195 | 392.1 | 1E-105 | 97 | -10.596257 | 255.680928 | 0.085366 | 279.6951 |
| 22 | c179745_g1_i1 | Maltase A1-like | XP_022821249.1 | 8E-18 | 239 | 373.6 | 8E-100 | 79 | 12.291215 | 233.955426 | 11.63333 | 256.375 |
| 23 | c155555_g3_i1 | Pancreatic triacylglycerol lipase-like | XP_021186847.1 | 3.31E-15 | 268 | 478 | 2E-131 | 88 | 15.228444 | 223.030038 | 14.35714 | 242.381 |
| 24 | c179009_g5_i3 | Usher syndrome type-1G protein homolog | XP_022831113.1 | 1.52E-16 | 329 | 597 | 4E-167 | 95 | 13.064535 | 196.884655 | 12.33333 | 215.1795 |
| 25 | c173690_g1_i1 | UDP-N-acetylglucosamine pyrophosphorylase | ACN29686.1 | 1.51E-15 |  |  |  |  | 8.950859 | 186.088327 | 8.681818 | 208.9091 |
| 26 | c179431_g3_i5 | Epoxide hydrolase 4-like isoform X1 | haw:110372229 | 5.2E-14 | 330 | 400.2 | 4E-108 | 88 | 30.045959 | 156.130991 | 28.84615 | 173.6154 |
| 27 | c25039_g1_i1 | Esterase E4-like | XP_022816644.1 | 1.63E-15 | 3341 | 2134 | 0 | 88 | 13.725186 | 143.742428 | 12.94937 | 156.9367 |
| 28 | c142545_g1_i1 | Probable peroxisomal acyl-coenzyme A oxidase 1 | XP_022821956.1 | 8.02E-16 | 2765 | 2752.6 | 0 | 86 | 9.904547 | 143.285796 | 9.376068 | 157.1538 |
| 29 | c159815_g1_i1 | Golgin subfamily A member 6-like protein | XM_022974360 | 1.41E-13 | 491 | 959.9 | 4E-276 | 97 | 9.737439 | 141.233628 | 9.320755 | 155.7925 |
| 30 | c145372_g1_i1 | Golgin subfamily A member 6-like protein 22 isoform X1 | XP_022830127.1 | 1.3E-12 | 175 | 361.3 | 3E-96 | 94 | 12.548782 | 138.837547 | 11.85542 | 149.759 |
| 31 | c177574_g1_i1 | Elongation factor Tu-like | XM_022981542 | 1.24E-11 | 464 | 895.6 | 8E-257 | 95 | 18.808814 | 130.083330 | 18 | 143.75 |
| 32 | c152136_g2_i5 | Balbiani ring protein 3-like isoform X1 | XP_022829635.1 | 7.56E-15 | 601 | 381.7 | 1E-102 | 91 | 5.945327 | 130.002637 | 5.586207 | 141.3103 |
| 33 | c172505_g1_i1 | Carbonyl reductase [NADPH] 1-like | haw:110374859 | 2.25E-11 | 241 | 490 | 5E-135 | 95 | 15.103443 | 122.378423 | 14.75 | 137.8333 |
| 34 | c181344_g4_i1 | Glutathione S-transferase epsilon 5 | AIH07580.1 | 3.16E-13 | 230 | 420.6 | 4E-114 | 93 | 20.090187 | 122.019336 | 19 | 133.619 |
| 35 | c180508_g2_i2 | Transmembrane protease serine 9-like | XP_022827086.1 | 2.78E-15 | 336 | 335.1 | 4E-88 | 89 | 6.378148 | 119.566918 | 5.992055 | 129.8162 |
| 36 | c177328_g1_i1 | Brachyurin-like | XP_022826482.1 | 1.22E-14 | 491 | 908.3 | 1E-260 | 90 | 19.747259 | 107.983096 | 18.5737 | 117.4014 |
| 37 | c172846_g1_i1 | Glucose 1-dehydrogenase-like | XM_022978640 | 2.74E-13 | 1937 | 817.8 | 2E-233 | 92 | 12.663579 | 96.853167 | 11.96703 | 105.6703 |
| 38 | c178372_g11_i1 | GDP-mannose 4,6 dehydratase | XP_022826969.1 | 7.1E-13 | 184 | 332 | 2E-87 | 83 | 10.613396 | 80.725006 | 10.0625 | 88.5125 |
| 39 | c214749_g1_i1 | Venom serine protease-like isoform X1 | XP_022818725.1 | 3.46E-13 | 613 | 646 | 1E-181 | 97 | -44.401305 | 77.125263 | 0.021277 | 83.88146 |
| 40 | c143838_g1_i1 | Transmembrane protease serine 9-like | XP_022827086.1 | 2.23E-12 | 311 | 578.9 | 1E-161 | 87 | 13.462851 | 75.310428 | 12.19275 | 77.03435 |
| 41 | c170749_g1_i1 | Cytochrome b561 domain-containing protein 1 | haw:110376488 | 1.15E-12 | 1087 | 1627.5 | 0 | 88 | 5.912922 | 73.343911 | 5.558824 | 79.72794 |
| 42 | c174289_g1_i1 | Grpe protein homolog, mitochondrial-like | XP_022835030.1 | 6.54E-12 | 534 | 887.9 | 5E-254 | 96 | 14.136619 | 72.240461 | 13.26471 | 78.27941 |
| 43 | c187955_g2_i1 | Luciferin 4-monooxygenase-like isoform X1 | XP_022831886.1 | 7.15E-12 | 525 | 609 | 2E-170 | 91 | 9.027991 | 71.126189 | 8.372603 | 75.25753 |
| 44 | c176762_g2_i1 | Collagenase-like | XP_022831349.1 | 2.88E-12 | 292 | 537.7 | 3E-149 | 92 | 9.775177 | 65.125330 | 9.221519 | 71.02532 |
| 45 | c183187_g4_i1 | Probable U3 small nucleolar RNA-associated protein 11 | XP_022829581.1 | 1.65E-09 | 302 | 589.3 | 7E-165 | 98 | 11.053576 | 64.663222 | 11 | 74.35714 |
| 46 | c187782_g5_i2 | Vanin-like protein 2 | XM_022975865 | 5.66E-12 |  |  |  |  | -74.283519 | 59.431834 | 0.013216 | 64.57269 |
| 47 | c189873_g1_i1 | Esterase FE4-like | XP_022821095.1 | 6.64E-12 | 335 | 644.4 | 2E-181 | 91 | 7.538110 | 58.385096 | 7.123077 | 63.87692 |
| 48 | c183854_g2_i3 | Cholinesterase 2-like | XP_022817753.1 | 9.25E-12 | 557 | 1064.3 | 2E-307 | 94 | 6.615502 | 55.832865 | 6.240506 | 60.9557 |
| 49 | c158284_g1_i1 | Transmembrane protease serine 9-like | XP_022827086.1 | 8.87E-12 | 313 | 423.7 | 6E-115 | 66 | 8.060805 | 53.049185 | 7.545528 | 57.23634 |
| 50 | c177568_g1_i1 | Coatomer subunit epsilon | XP_022824606.1 | 2.39E-11 | 547 | 1049.3 | 5E-303 | 92 | 7.490707 | 51.260328 | 7.035714 | 55.6039 |
| 51 | c188384_g2_i5 | Dosage compensation regulator isoform X1 | XP_022827412.1 | 1.75E-10 | 362 | 704.5 | 2E-199 | 95 | 7.146479 | 48.144619 | 6.823529 | 53.11765 |
| 52 | c186477_g5_i1 | ER membrane protein complex subunit 6 | XP_022827452.1 | 6.84E-10 | 1014 | 956.1 | 5E-275 | 89 | 8.028456 | 47.209453 | 7.626667 | 51.74667 |
| 53 | c187474_g1_i1 | Presequence protease, mitochondrial | XP_022816885.1 | 1.3E-10 | 2711 | 147 | 1E-30 | 90 | 8.092411 | 44.589554 | 7.6 | 48.575 |
| 54 | c157208_g1_i1 | Androgen-dependent TFPI-regulating protein-like | XP_022820828.1 | 9.14E-11 | 326 | 648.7 | 3E-182 | 97 | 5.655072 | 43.116741 | 5.308943 | 46.72764 |
| 55 | c183552_g1_i3 | Cytochrome P450 CYP306A1 | ACM45975.1 | 3.28E-10 | 494 | 330.5 | 1E-86 | 72 | 7.599503 | 40.603744 | 7.1375 | 44.175 |
| 56 | c188868_g1_i2 | Protein transport protein Sec31A isoform X3 | XP_022822559.1 | 1.27E-09 | 301 | 585.9 | 2E-163 | 95 | 6.120695 | 32.775211 | 5.754386 | 35.69006 |
| 57 | c130189_g1_i3 | Glyoxylate reductase/hydroxypyruvate reductase-like | XP_022823204.1 | 2.08E-09 | 454 | 888.6 | 2E-254 | 93 | 5.466897 | 32.550352 | 5.148515 | 35.46535 |
| 58 | c55326_g1_i1 | Fibrinogen silencer-binding protein-like | XP_022834579.1 | 0.000834 | 551 | 1077.4 | 0 | 97 | 249.463066 | 30.273239 | 259 | 36 |
| 59 | c182737_g1_i2 | Retinol dehydrogenase 11-like | XP_022818680.1 | 3.72E-05 | 1884 | 1162.7 | 0 | 86 | 205.617285 | 19.628810 | 192.25 | 21.16667 |
| 60 | c155989_g1_i1 | Cubilin-like | XP_022829063.1 | 3.41E-06 | 609 | 1169.5 | 0 | 90 | 89.728998 | 14.559030 | 85.63636 | 16.09091 |
| 61 | c184650_g2_i2 | Reverse ribonuclease integrase, partial | KMQ85528.1 | 4.62E-05 | 517 | 504.6 | 3E-139 | 66 | 133.570224 | 12.787038 | 119.5 | 13.25 |
| 62 | c180292_g9_i4 | SID1 transmembrane family member 2-like | XP_022814675.1 | 1.91E-05 | 319 | 524.6 | 5E-145 | 90 | -11.668695 | 11.765021 | 0.081818 | 12.79091 |
| 63 | c177662_g1_i1 | Pancreatic triacylglycerol lipase-like | XP_022831990.1 | 7.42E-06 | 721 | 1365.1 | 0 | 92 | -11.991366 | 11.688653 | 0.078267 | 12.70219 |
| 64 | c185217_g6_i1 | Cytosolic 10-formyltetrahydrofolate dehydrogenase | XP_022831585.1 | 6.17E-05 | 4882 | 157.5 | 2E-34 | 93 | 60.345831 | 9.115297 | 56.92975 | 9.96281 |
| 65 | c170666_g1_i1 | 3-ketodihydrosphingosine reductase | XP_022828258.1 | 8.33E-05 | 908 | 634.4 | 3E-178 | 86 | -33.447863 | 8.789480 | 0.027237 | 9.549935 |
| 66 | c174187_g1_i1 | 39S ribosomal protein L16, mitochondrial | XP_022819705.1 | 0.000284 | 392 | 681.8 | 3E-192 | 82 | -6.921639 | 7.640454 | 0.134904 | 8.280514 |
| 67 | c98457_g2_i1 | Myosin heavy chain, muscle isoform X38 | XP_022837940.1 | 0.002138 | 590 | 535 | 2E-148 | 92 | 42.399187 | 7.615262 | 39.95283 | 8.235849 |
| 68 | c181305_g3_i1 | 2-acylglycerol O-acyltransferase 1-like | XP_022825607.1 | 0.000353 | 146 | 262.7 | 3E-66 | 85 | -7.462295 | 7.131222 | 0.125632 | 7.766065 |
| 69 | c176152_g5_i4 | Eukaryotic translation initiation factor 4E-like | XP_022816684.1 | 0.002247 | 310 | 153.3 | 2E-33 | 98 | -21.186798 | 5.818329 | 0.042105 | 6.342105 |
| 70 | c174059_g1_i2 | Putative inorganic phosphate cotransporter | XP_022837725.1 | 0.001672 | 286 | 576.6 | 1E-160 | 96 | -14.220944 | 5.768003 | 0.066528 | 6.276507 |
| 71 | c99163_g1_i1 | Myosin heavy chain, muscle | KOB77375.1 | 0.007992 | 235 | 446 | 1E-121 | 89 | 39.010370 | 5.346504 | 37.17021 | 5.893617 |
| 72 | c172390_g1_i2 | Pancreatic lipase-related protein 2-like | XP_022833584.1 | 0.003155 | 1175 | 568 | 0 | 94 | -7.090387 | 5.323064 | 0.131545 | 5.747126 |
| 73 | c166571_g1_i1 | Elongation of very long chain fatty acids protein 7-like | XP_022817724.1 | 0.005744 | 410 | 785 | 1E-223 | 96 | 14.623867 | -5.270324 | 13.75046 | 0.205829 |
| 74 | c181556_g2_i1 | Lachesin-like isoform X1 | XP_022826094.1 | 0.002864 | 359 | 713.4 | 5E-202 | 95 | -39.030882 | -5.282610 | 0.024026 | 0.206494 |
| 75 | c182447_g6_i1 | Cytochrome P450 CYP4G75 | AGO62001.1 | 0.001181 | 260 | 403.3 | 5E-109 | 90 | 13.545678 | -6.009083 | 12.76969 | 0.181769 |
| 76 | c167862_g1_i1 | Protein THEM6-like | XP_022816629.1 | 0.00294 | 228 | 449.1 | 2E-122 | 96 | 10.729503 | -6.033243 | 10.06044 | 0.178571 |
| 77 | c186135_g4_i3 | Homeobox protein unc-4 isoform X1 | XP_022830744.1 | 0.000313 | 549 | 958.4 | 1E-275 | 84 | 5.514898 | -8.816114 | 5.18797 | 0.120301 |
| 78 | c180993_g6_i6 | Meiosis-specific with OB domain-containing protein | XP_022829909.1 | 0.000121 | 400 | 818.5 | 2E-233 | 100 | -68.513559 | -9.068597 | 0.013514 | 0.119369 |
| 79 | c162995_g1_i3 | Larval cuticle protein 1-like | XP_022816717.1 | 1.1E-05 | 522 | 954.9 | 2E-274 | 87 | 5.208805 | -11.141481 | 4.89646 | 0.097559 |
| 80 | c179887_g6_i5 | Retinol dehydrogenase 11-like | XP_022824576.1 | 3.95E-05 | 628 | 592.8 | 2E-165 | 74 | 8.271275 | -11.215356 | 7.786364 | 0.097727 |
| 81 | c37997_g1_i1 | Elongation of very long chain fatty acids protein | XP_022816385.1 | 0.000333 | 372 | 427.9 | 5E-116 | 82 | 56.710977 | -21.289064 | 53.8 | 0.047059 |
| 82 | c177235_g2_i1 | Putative UDP-glucuronosyltransferase ugt-58 | XP_022817908.1 | 4.84E-08 | 557 | 1122.8 | 0 | 100 | 10.738029 | -28.117291 | 10.10882 | 0.038235 |
| 83 | c180258_g1_i4 | Obscurin isoform X6 | XP_022821457.1 | 1.11E-09 | 9533 | 70.9 | 9E-09 | 54 | -5.336863 | -39.603418 | 0.176598 | 0.027086 |
| 84 | c180836_g3_i3 | Attacin-like | XP_022837464.1 | 4.14E-09 | 328 | 540.8 | 4E-150 | 82 | 6.405328 | -41.012255 | 6.019308 | 0.026549 |
| 85 | c180588_g2_i1 | Ecdysteroid-regulated 16 kda protein | XP_022826985.1 | 2.08E-12 | 484 | 884.8 | 2E-253 | 87 | -7.703802 | -62.634691 | 0.122186 | 0.017399 |
| 86 | c185248_g1_i2 | Lysosomal beta-N-acetylglucosaminidase | BAN29056.1 | 1.08E-12 | 252 | 498.4 | 2E-137 | 98 | -6.709779 | -66.560536 | 0.140399 | 0.016384 |
| 87 | c182570_g3_i3 | Twitchin [Helicoverpa armigera] | XP_021187044.1 | 6.59E-13 | 539 | 1099 | 0 | 98 | -5.389098 | -73.954915 | 0.174182 | 0.014645 |
| 88 | c187863_g3_i2 | Peptidoglycan-recognition protein LB-like | XP_022831852.1 | 1.82E-08 | 381 | 436.8 | 9E-119 | 82 | -5.833859 | -75.307418 | 0.159538 | 0.013873 |
| 89 | c182172_g4_i2 | Mucin-2-like | XP_022832666.1 | 2.77E-13 | 422 | 795 | 1E-226 | 90 | -9.075581 | -76.869373 | 0.103779 | 0.014195 |
| 90 | c186831_g1_i1 | Myrosinase 1-like isoform X1 | XP_022818244.1 | 1.28E-12 | 610 | 508.1 | 2E-140 | 82 | -11.040426 | -85.600467 | 0.085315 | 0.012587 |
| 91 | c180757_g2_i1 | Elongation of very long chain fatty acids protein | XP_022816380.1 | 3.13E-12 | 548 | 967.6 | 2E-278 | 85 | -10.834959 | -91.790428 | 0.086556 | 0.011664 |
| 92 | c138132_g1_i1 | Glucose-1-phosphatase-like | XM_022968239 | 1.61E-14 | 549 | 1052.7 | 4E-304 | 92 | -5.848531 | -103.038023 | 0.160844 | 0.01055 |
| 93 | c175570_g1_i3 | Sialin | XP_022822670.1 | 8.04E-11 | 528 | 594.7 | 6E-166 | 98 | -5.433577 | -111.887932 | 0.173358 | 0.009124 |
| 94 | c176083_g2_i2 | Zonadhesin-like | XP_022826186.1 | 2.43E-14 | 1188 | 684.9 | 8E-193 | 42 | -6.091510 | -112.409499 | 0.154327 | 0.009684 |
| 95 | c186112_g2_i1 | Luciferin 4-monooxygenase-like | XP_022834772.1 | 9.84E-14 | 148 | 291.2 | 3E-75 | 91 | -12.774137 | -112.963713 | 0.073788 | 0.009438 |
| 96 | c182396_g1_i2 | Actin cytoskeleton-regulatory complex protein PAN1-like | XP_022825631.1 | 3.27E-13 | 525 | 752.7 | 7E-214 | 94 | -6.856293 | -114.124304 | 0.137286 | 0.009615 |
| 97 | c177048_g3_i9 | Protein FAM13A isoform X3 | XP_022816420.1 | 4.3E-15 | 927 | 1819.3 | 0 | 96 | -6.232104 | -133.130451 | 0.151316 | 0.008333 |
| 98 | c184148_g1_i7 | Solute carrier family 22 member 5-like isoform X2 | XP_022834032.1 | 8.49E-15 | 586 | 1223 | 0 | 99 | -6.891191 | -142.085380 | 0.136861 | 0.007755 |
| 99 | c185017_g2_i1 | Odorant binding protein 17 | AKT26495.1 | 1.76E-16 | 548 | 1065.1 | 9E-308 | 94 | -18.851103 | -171.860192 | 0.04971 | 0.00628 |
| 100 | c168383_g1_i1 | Odorant binding protein 25 | AKT26502.1 | 8.61E-14 | 531 | 985.7 | 8E-284 | 91 | 6.026591 | -201.034409 | 5.666842 | 0.005263 |
| 101 | c151342_g1_i1 | Diapausin precursor | ABU96713.1 | 1.03E-15 | 347 | 662.9 | 2E-186 | 99 | -10.583129 | -208.991960 | 0.088008 | 0.005061 |
| 102 | c176853_g1_i1 | Myosin-7 | XP_022826595.1 | 9.37E-18 | 114 | 199.5 | 1E-47 | 98 | -15.421761 | -229.313170 | 0.061084 | 0.004721 |
| 103 | c183805_g5_i1 | Transmembrane protease serine 9-like | XP_022827091.1 | 4.73E-18 | 496 | 952.2 | 9E-274 | 90 | -14.234498 | -252.797522 | 0.065919 | 0.004274 |
| 104 | c180815_g1_i1 | Putative phosphatidate phosphatase | XP_022816068.1 | 1.81E-17 | 1856 | 1295.6 | 0 | 86 | -40.293601 | -263.005899 | 0.023356 | 0.004161 |
| 105 | c183748_g1_i1 | Lipase 1-like | XP_022818761.1 | 5.38E-15 | 1014 | 1986.5 | 0 | 94 | -6.945573 | -304.431349 | 0.135394 | 0.003198 |
| 106 | c177952_g4_i2 | Lymphocyte antigen 75-like | XP_022816384.1 | 2.21E-18 | 975 | 710.2 | 0 | 80 | -19.585158 | -329.554632 | 0.047961 | 0.003243 |
| 107 | c178940_g11_i3 | Sideroflexin-2 | XP_022828273.1 | 2.02E-19 | 196 | 166.8 | 8E-38 | 88 | -46.901622 | -342.472948 | 0.020097 | 0.003159 |
| 108 | c181642_g4_i1 | UDP-glucuronosyltransferase 2B15-like | XP_022815547.1 | 3.16E-16 | 544 | 202.2 | 9E-49 | 91 | 6.355989 | -355.777359 | 5.98519 | 0.00322 |
| 109 | c183693_g1_i1 | Gastrula zinc finger protein xlcgf17.1-like | XP_022827449.1 | 2.91E-13 | 1302 | 2307.7 | 0 | 96 | -14.927111 | -374.803335 | 0.063119 | 0.002475 |
| 110 | c180865_g3_i2 | MARVEL domain-containing protein 1-like | XM_022965385 | 3.62E-14 | 1202 | 1107 | 0 | 92 | -17.209522 | -463.954716 | 0.054273 | 0.002309 |
| 111 | c185110_g6_i1 | Facilitated trehalose transporter Tret1-like | XP_022815344.1 | 6.82E-16 | 501 | 1013.1 | 5E-292 | 99 | -18.438567 | -465.654329 | 0.05102 | 0.002319 |
| 112 | c185794_g2_i2 | Organic cation transporter protein-like | XP_022828278.1 | 1.13E-17 | 545 | 1024.2 | 2E-295 | 89 | -9.073745 | -494.214062 | 0.103538 | 0.002235 |
| 113 | c174088_g2_i1 | Transmembrane protease serine 9-like | XP_022827088.1 | 4.77E-20 | 449 | 755 | 1E-214 | 86 | -19.381141 | -565.742669 | 0.048211 | 0.001877 |
| 114 | c174521_g1_i1 | Aldo-keto reductase AKR2E4-like isoform X2 | XP_022817297.1 | 3.42E-15 | 630 | 1113.6 | 0 | 93 | -26.329947 | -588.043801 | 0.035285 | 0.001604 |
| 115 | c176239_g13_i1 | Transmembrane protease serine 9-like | XP_022826383.1 | 3.46E-20 | 583 | 1132.1 | 0 | 93 | -17.145206 | -724.742176 | 0.054824 | 0.001449 |
| 116 | c154439_g1_i1 | Larval cuticle protein LCP-30-like | XP_022822471.1 | 3.22E-22 | 295 | 572 | 2E-159 | 92 | -6.292687 | -745.883851 | 0.149475 | 0.00146 |
| 117 | c173756_g5_i1 | Gloverin-like | XP_022837404.1 | 1.54E-21 | 263 | 224.6 | 6E-55 | 61 | -61.023147 | -806.960591 | 0.015361 | 0.001327 |
| 118 | c181312_g4_i1 | Trypsin, alkaline C-like | XP_022815738.1 | 8.78E-23 | 1964 | 126.3 | 9E-26 | 100 | -5.980899 | -1127.841097 | 0.156322 | 0.000938 |
| 119 | c187398_g1_i1 | Leukocyte elastase inhibitor-like | haw:110382605 | 8.55E-24 | 2439 | 72.4 | 4E-09 | 62 | -6.664743 | -4625.155673 | 0.141379 | 0.000185 |
